# Longitudinal Neuropathological Consequences of Extracranial Radiation Therapy in Mice

**DOI:** 10.3390/ijms25115731

**Published:** 2024-05-24

**Authors:** Kimberly Demos-Davies, Jessica Lawrence, Jessica Coffey, Amy Morgan, Clara Ferreira, Luke H. Hoeppner, Davis Seelig

**Affiliations:** 1 Department of Veterinary Clinical Sciences, University of Minnesota College of Veterinary Medicine, Saint Paul, MN 55108, USA; jlawrence@ucdavis.edu (J.L.); jkcoffey@umn.edu (J.C.); newl0026@d.umn.edu (A.M.); dseelig@umn.edu (D.S.); 2 Masonic Cancer Center, University of Minnesota, Minneapolis, MN 55455, USA; hoepp005@umn.edu; 3 Department of Radiation Oncology, University of Minnesota Medical School, Minneapolis, MN 55455, USA; cferreir@umn.edu; 4 The Hormel Institute, University of Minnesota, 801 16th Ave NE, Austin, MN 55912, USA

**Keywords:** cancer-related cognitive impairment CRCI, neuropathology, cancer treatment, SKH1 mice, radiation-related cognitive impairment

## Abstract

Cancer-related cognitive impairment (CRCI) is a consequence of chemotherapy and extracranial radiation therapy (ECRT). Our prior work demonstrated gliosis in the brain following ECRT in SKH1 mice. The signals that induce gliosis were unclear. Right hindlimb skin from SKH1 mice was treated with 20 Gy or 30 Gy to induce subclinical or clinical dermatitis, respectively. Mice were euthanized at 6 h, 24 h, 5 days, 12 days, and 25 days post irradiation, and the brain, thoracic spinal cord, and skin were collected. The brains were harvested for spatial proteomics, immunohistochemistry, Nanostring nCounter^®^ glial profiling, and neuroinflammation gene panels. The thoracic spinal cords were evaluated by immunohistochemistry. Radiation injury to the skin was evaluated by histology. The genes associated with neurotransmission, glial cell activation, innate immune signaling, cell signal transduction, and cancer were differentially expressed in the brains from mice treated with ECRT compared to the controls. Dose-dependent increases in neuroinflammatory-associated and neurodegenerative-disease-associated proteins were measured in the brains from ECRT-treated mice. Histologic changes in the ECRT-treated mice included acute dermatitis within the irradiated skin of the hindlimb and astrocyte activation within the thoracic spinal cord. Collectively, these findings highlight indirect neuronal transmission and glial cell activation in the pathogenesis of ECRT-related CRCI, providing possible signaling pathways for mitigation strategies.

## 1. Introduction

Cancer patients often suffer from a syndrome of neurocognitive dysfunction termed cancer-related cognitive impairment (CRCI) [1,2]. Chemotherapy and extracranial radiation therapy (ECRT) have been associated with long-standing CRCI [1,2,3]. CRCI affects the quality of life by decreasing functional independence in an estimated 75% of cancer survivors [4,5]. CRCI has been reported in cancer survivors following treatment for solid tumors including breast, lung, intestinal, ovarian, prostate, and testicular tumors [1,2,4,5]. Women with breast cancer are the most well documented to suffer from CRCI and report cognitive impairment up to 10 years following treatment with chemotherapy and/or radiation therapy [1,6].

Breast cancer survivors treated with extracranial radiation therapy (ECRT) alone have shown cognitive impairment months to years following treatment [7,8,9,10,11]. ECRT is a standard-of-care treatment for multiple cancers; indeed, over 60% of all newly diagnosed cancer patients or over 12 million people will receive ECRT at some point during their treatment [12]. Clinical signs of CRCI following ECRT as a sole treatment include memory loss, fatigue, and impairment of concentration that can be sustained for years after the completion of treatment [1,2]. While the association between radiation therapy and CRCI is strong, the mechanism by which ECRT causes CRCI is unknown which makes prevention and treatment strategies of ECRT-induced CRCI challenging [4].

The effects of direct radiation therapy on the normal brain are well known and include brain damage and neuroinflammation that lead to neurocognitive decline [13,14,15]. However, less is understood about the effects of ECRT-induced injury on the brain. One proposed mechanism is that radiation induces inflammation at the irradiated site creating proinflammatory cytokines in circulation leading to cognitive deficits via neuroinflammation in cancer patients [1,3,8,16]. One study demonstrated that memory deficits in breast cancer patients up to 7 months following treatment with ECRT was partially mediated by elevated plasma IL-6 levels [16]. Several studies have shown that breast cancer patients who received ECRT have subsequent impairment in multiple cognitive domains, including complex cognition, attention, memory, and executive function [1,2,7]. According to the 2022 GLOBOCAN cancer burden survey, there were an estimated 20 million new cancer cases worldwide in 2022, and this number is increasing every year [17,18]. With the increase in cancer incidence, there will be an increased number of cancer survivors at risk for ECRT-induced CRCI. It is imperative to further understand the mechanisms of ECRT-induced CRCI to shed light on mitigation strategies to improve cancer survivors’ quality of life.

Limited in-vivo studies have shown that ECRT in mouse models leads to neuroinflammation evident by the activation of microglia and astrocytes [1,2,3]. The severity of this neuroinflammation is comparable to mice exposed to chemotherapy agents of methotrexate or doxorubicin [1,2,3]. Our prior work showed that SKH1 mice treated with ECRT caused focal dermatitis and hippocampal dependent memory deficits comparable to SKH1 mice treated with doxorubicin, a potent broad-spectrum anti-cancer drug known to induce cognitive deficits [3,19]. There is a limited understanding as to the clinical cognitive impact of ECRT and the mechanism(s) that link CRCI and radiation prescribed to anatomic sites distant from the brain. We previously characterized an SKH1 mouse model of ECRT-induced CRCI, and this model allows us to investigate the crucial mechanisms by which ECRT triggers neuroinflammatory pathology [3,20]. Because breast cancer survivors are over-represented in populations with ECRT-induced CRCI, acute dermatitis is the most common radiation-induced acute adverse event in breast cancer patients, and our prior work demonstrating the occurrence of CRCI following radiation in SKH1 mice, our studies utilize this strain to examine the effects of ECRT on the central nervous system over time [1,2,3,7,16,21]. SKH1 mice are outbred, immunologically competent, and commonly used for dermatologic studies due to the similarities in their skin composition with that of humans [12,22]. In this study, we evaluated brain and spinal cord changes following two distinct radiation doses capable of causing subclinical and clinically evident dermatitis, respectively. 

## 2. Results

### 2.1. Extracranial Radiation Therapy Causes Acute Toxicity in the Skin of Mice

**Figure 1:**
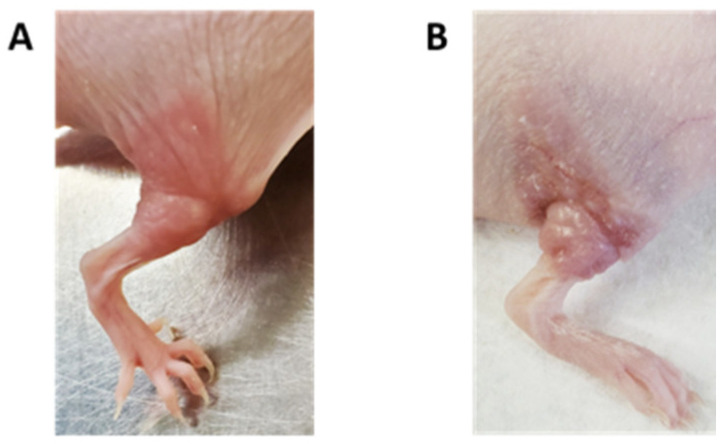
Acute skin radiation toxicity in mice treated with hindlimb radiation. (**A**) Representative image of the irradiated right hindlimb from a mouse treated with 30 Gy. Focal erythema and mild dermatitis started on day 10 post treatment. (**B**) Representative image of severe, ulcerative dermatitis within the irradiated right hindlimb of a mouse treated with 30 Gy. Changes shown developed on day 12 post treatment.

**Figure 2:**
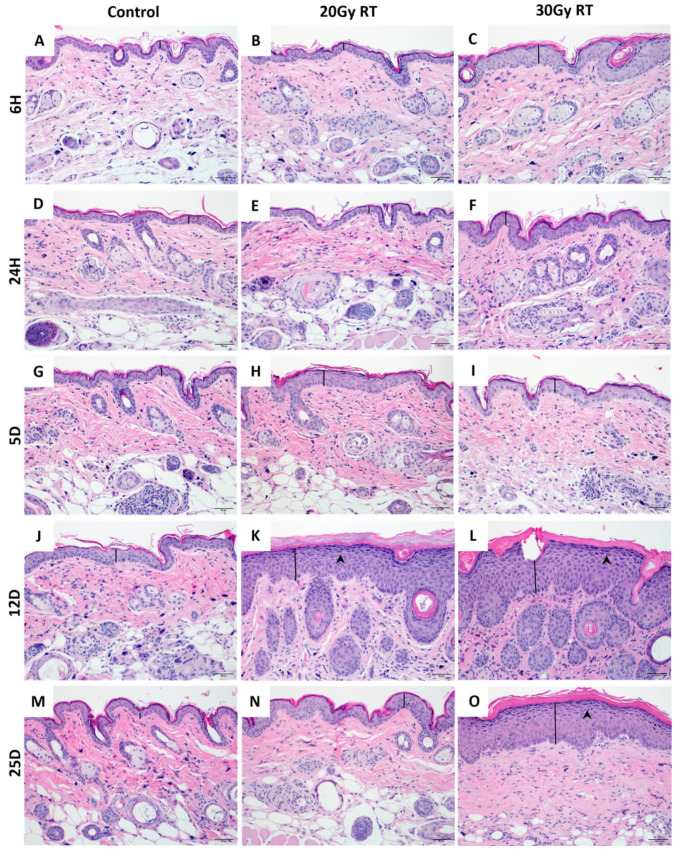
Histologic evidence of radiation-induced dermatitis in the hindlimb skin of SKH-1 mice at various timepoints after irradiation. Control skin was obtained from unirradiated right hindlimb skin from age-matched SKH-1 mice at the following timepoints: 6 h (**A**), 24 h (**D**), 5 days (**G**), 12 days (**J**), and 25 days (**M**). Representative images from skin on the right hindlimb following 20 Gy at 6 h (**B**), 24 h (**E**), 5 days (**H**), 12 days (**K**), and 25 days (**N**) post radiation treatment. Representative images from skin on the right hindlimb following 30 Gy at 6 h (**C**), 24 h (**F**), 5 days (**I**), 12 days (**L**), and 25 days (**O**) post radiation treatment. Hyperpigmentation (arrow heads) and epidermal thickening (black line) was evident following 20 Gy at 12 days (**K**) and following 30 Gy at 12 days (**L**) and 25 days (**O**). Images shown at 200×. *n* = 4; Control = control mice group; 20 Gy RT = mice treated with 20 Gy to hindlimb; 30 Gy RT = mice treated with 30 Gy to hindlimb. H&E.

Acute radiation-induced dermatitis is a common side effect of RT, affecting up to 90% of cancer patients who undergo radiation therapy treatment [23,24]. Cutaneous erythema was visible in mice treated with 30 Gy starting at day 10 (Figure 1A) with progression to confluent moist dermatitis or ulceration by day 12 (Figure 1B). To characterize the severity of radiation dermatitis, the irradiated skin in each mouse was evaluated for histological changes over time. Mice receiving either 20 Gy or 30 Gy radiation to their hindlimb had significant pathological dermal and epidermal changes (Figure 2). Marked histological changes in all irradiated mice including thickening, parakeratosis, and intracellular edema in the epidermis (Figure 2K,L) were compared to the control mice on day 12. The skin from 20 Gy and 30 Gy treated mice on day 12 showed dermal hyperpigmentation (Figure 2K,L). Histologic changes were more severe and protracted in the mice treated with 30 Gy compared to 20 Gy (Figure 2K,L,N,O). 

### 2.2. Extracranial Radiation Increases Activation of Astrocytes in the Thoracic Spinal Cord of Mice

**Figure 3:**
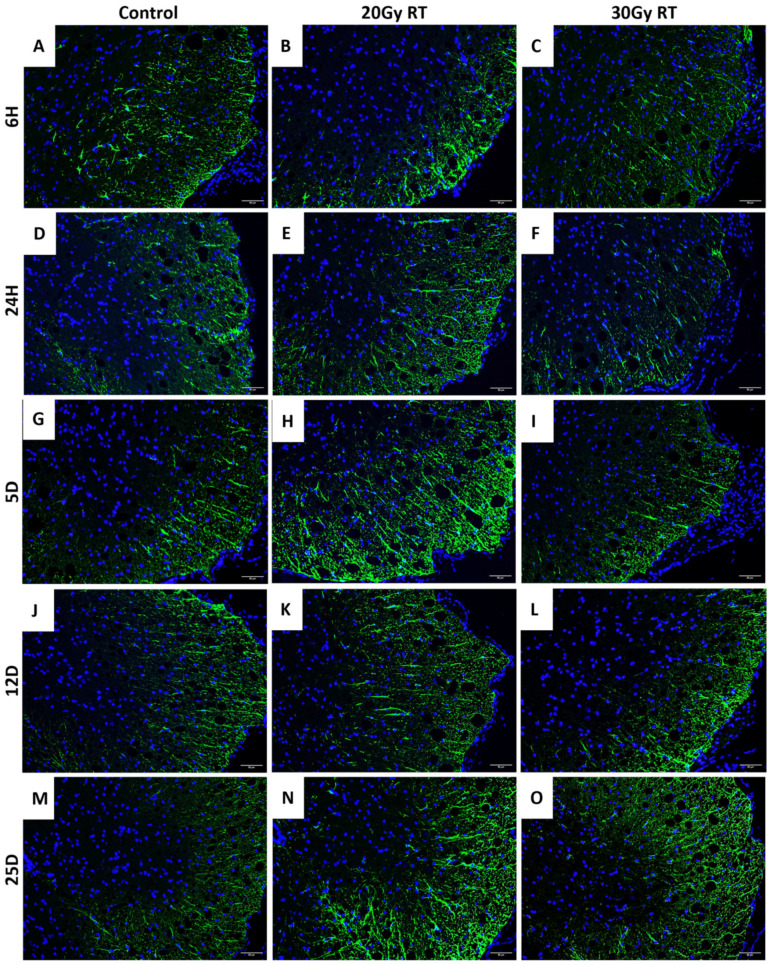
The expression of GFAP within the thoracic spinal cord after ECRT in mice. Representative images depict activated astrocytes (GFAP+ cells) in the thoracic spinal cord from control mice at 6 h (**A**), 24 h (**D**), 5 days (**G**), 12 days (**J**), and 25 days (**M**). Representative images depict activated astrocytes (GFAP+ cells) in the thoracic spinal cord from mice treated with 20 Gy at 6 h (**B**), 24 h (**E**), 5 days (**H**), 12 days (**K**), and 25 days (**N**) post radiation treatment. Representative images depict activated astrocytes (GFAP+ cells) in the thoracic spinal cord from mice treated with 30 Gy at 6 h (**C**), 24 h (**F**), 5 days (**I**), 12 days (**L**), and 25 days (**O**) post radiation treatment. Images shown are at 200×. Control = control mice group; 20 Gy RT = mice treated with 20 Gy to hindlimb; 30 Gy RT = mice treated with 30 Gy to hindlimb.

**Figure 4:**
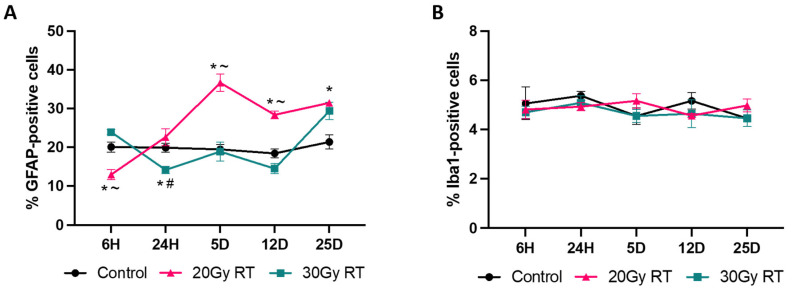
The expression changes in GFAP and Iba1 within the thoracic spinal cord after ECRT in mice. Line graph (**A**) depicting the percentage of astrocytes (GFAP+ cells) over time across treatment groups. Line graph (**B**) depicting the percentage of microglia (Iba1+ cells) over time across treatment groups. Data represent the mean and SEM (*n* = 4 mice from each group). * *p*-value < 0.05 compared to control group; # *p*-value < 0.05 compared to 20 Gy RT group; ~ *p*-value < 0.05 compared to 30 Gy RT group.

Previous studies have shown that ECRT causes increased activation of astrocytes and microglia in the brain of mice following treatment [1,3]. In our study, the right hindlimb was abducted from the body for treatment, and the lumbar spinal cord was actively shielded from the radiation field, confirmed by in vivo dosimetric measurements of the skin over the cord. The thoracic spinal cord was evaluated to assess if ECRT induced glial cell activation similar to that in the brain, or if neuroinflammation was limited to the brain. The mice treated with 20 Gy had a significantly reduced GFAP expression in the thoracic spinal cord 6 h post treatment (Figure 3B and Figure 4A) compared to the control mice (Figure 3A and Figure 4A). The mice treated with 20 Gy had a significant increase in activated astrocytes in the thoracic spinal cord 5 days (Figure 3H and Figure 4A), 12 days (Figure 3K and Figure 4A), and 25 days (Figure 3N and Figure 4A) post radiation treatment compared to the control mice (Figure 3G,J,M and Figure 4A). The mice treated with 30 Gy had a significantly reduced GFAP expression in the thoracic spinal cord 24 h post treatment (Figure 3F and Figure 4A) compared to the control mice (Figure 3D and Figure 4A). However, these mice subsequently had a significant increase in their GFAP expression in the thoracic spinal cord 25 days (Figure 3O and Figure 4A) post radiation treatment compared to the control mice (Figure 3M and Figure 4A). The mice treated with 20 Gy had a significant increase in activated astrocytes in the thoracic spinal cord 24 h (Figure 3E and Figure 4A), 5 days (Figure 3H and Figure 4A), and 12 days (Figure 3K and Figure 4A) compared to the mice treated with 30 Gy (Figure 3F,I,L and Figure 4A). There was no significant change in the microglial Iba1 expression between the groups throughout this study (Figure 4B). 

### 2.3. Extracranial Causes Significant Glial Cell Activation in the Striatum

**Figure 5:**
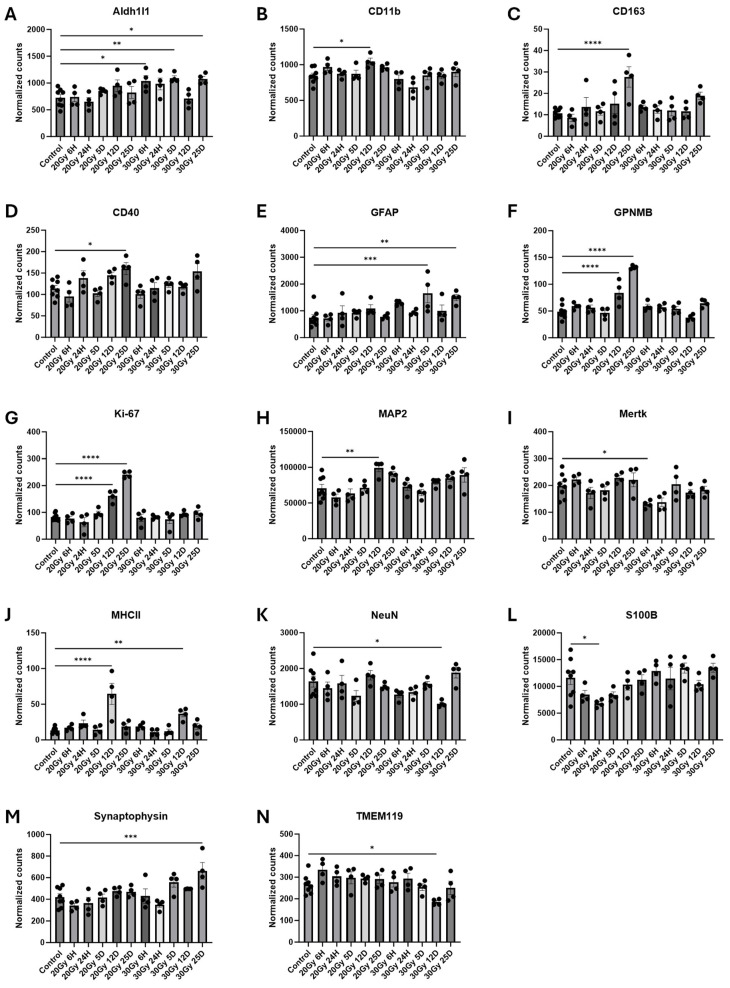
Glial cell activation in the striatum of ECRT-treated mice. Mice treated with 20 Gy demonstrated significant upregulation of CD11b (**B**), CD163 (**C**), CD40 (**D**), GPNMB (**F**), Ki-67 (**G**), MAP2 (**H**), and MHCII (**J**). Mice treated with 20 Gy demonstrated significant downregulation of S100B (**L**). Mice treated with 30 Gy demonstrated significant upregulation of Aldh1l1 (**A**), GFAP (**E**), MHCII (**J**), and Synaptophysin (**M**). Mice treated with 30 Gy demonstrated significant downregulation of Mertk (**I**), NeuN (**K**), and TMEM119 (**N**). Data represent the mean ± SEM and black dots represent individual values (*n* = 4–8 mice per group). Control = control mice that were euthanized at 6 h and 25 days. 20 Gy = mice treated with 20 Gy to hindlimb; 30 Gy = mice treated with 30 Gy to hindlimb. * *p* < 0.05; ** *p* < 0.01; *** *p* = 0.01–0.001; **** *p* < 0.001.

The striatum is involved in memory and learning which are two cognitive domains that are impaired in cancer survivors suffering from CRCI [3,25]. Since previous studies have shown increased expressions of the GFAP and IBA1 protein within the striatum of the mice after ECRT, we investigated the effect of ECRT on additional glial cell activation proteins within this region of the brain [1,3]. The mice treated with 20 Gy had significant upregulations of several proteins 12 days following treatment compared to the control mice, including CD11b (Table S1 and Figure 5B), GPNMB (Table S1 and Figure 5F), Ki-67 (Table S1 and Figure 5G), MAP2 (Table S1 and Figure 5H), and MHCII (Table S1 and Figure 5J). At 25 days post treatment, this 20 Gy group had upregulations of CD163 (Table S1 and Figure 5C), CD40 (Table S1 and Figure 5D), GPNMB (Table S1 and Figure 5F), and Ki-67 (Table S1 and Figure 5G) proteins compared to the control mice. Significantly decreased levels of the S100B (Table S1 and Figure 5L) protein were measured in the mice treated with 20 Gy compared to the controls 24 h post treatment. 

The mice treated with 30 Gy had a significant upregulation of Aldh1l1 (Table S1 and Figure 5A) protein and significantly decreased levels of the Mertk (Table S1 and Figure 5I) protein in their striatum compared to the control mice at 6 h post treatment. At 5 days post treatment, these mice had significant upregulations of the Aldh1l1 (Table S1 and Figure 5A) and GFAP (Table S1 and Figure 5E) proteins compared to the control mice. At 12 days post treatment, a significant upregulation of the MHCII (Table S1 and Figure 5J) protein and significant downregulations of the NeuN (Table S2 and Figure 5K) and TMEM119 (Table S1 and Figure 5N) proteins were measured in the striatum from the mice treated with 30 Gy compared to that from the control mice. Finally, at 25 days post 30 Gy, the murine striatum had significant upregulations of the Aldh1l1 (Table S1 and Figure 5A), GFAP (Table S1 and Figure 5E), and synaptophysin (Table S1 and Figure 5M) proteins compared to that of the control mice. 

### 2.4. Extracranial Causes Significant Glial Cell Activation in the Retrosplenial Cortex

**Figure 6:**
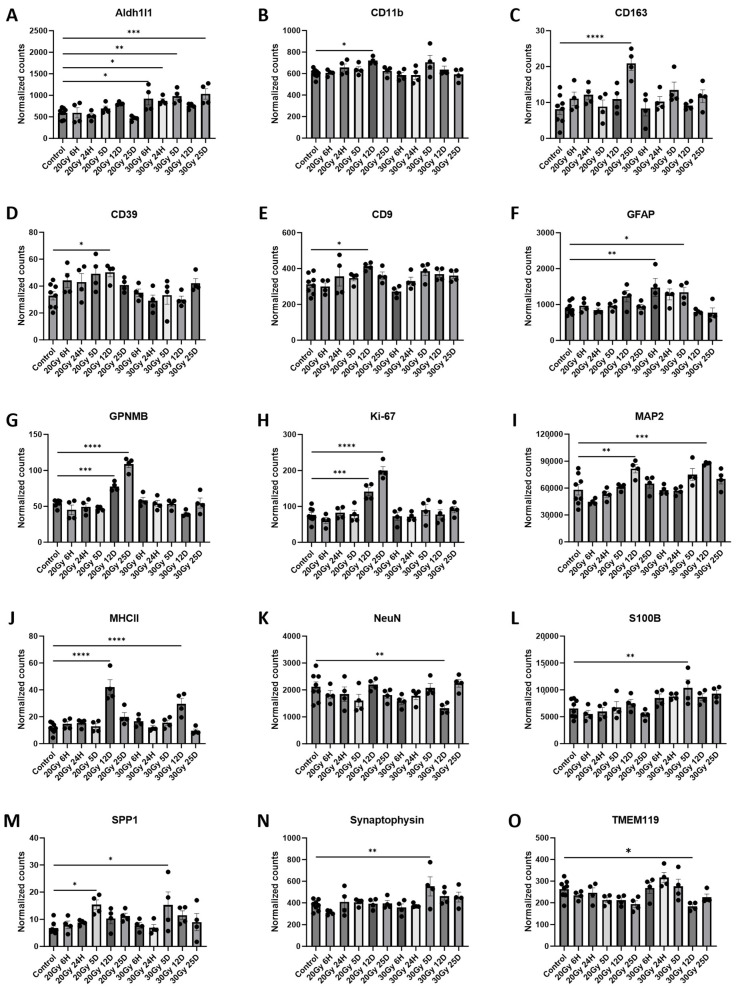
Glial cell activation in the retrosplenial cortex of ECRT-treated mice. Retrosplenial cortex from mice treated with 20 Gy demonstrated significant upregulation of CD11b (**B**), CD163 (**C**), CD39 (**D**), CD9 (**E**), GPNMB (**G**), Ki-67 (**H**), MAP2 (**I**), MHCII (**J**), and SPP1 (**M**). Mice treated with 30 Gy demonstrated significant upregulation of Aldh1l1 (**A**), GFAP (**F**), MAP2 (**I**), MHCII (**J**), S100B (**L**), SPP1 (**M**), and Synaptophysin (**N**). Mice treated with 30 Gy demonstrated significant downregulation of NeuN (**K**) and TMEM119 (**O**). Data represent the mean ± SEM and black dots represent individual values (*n* = 4–8 mice per group). Control = control mice that were euthanized at 6 h and 25 days. 20 Gy RT = mice treated with 20 Gy to hindlimb; 30 Gy RT = mice treated with 30 Gy to hindlimb. * *p* < 0.05; ** *p* < 0.01; *** *p* = 0.01–0.001; **** *p* < 0.001.

Upregulations of both the IBA1 and GFAP proteins in the caudal cortex, where the retrosplenial cortex is located, have been previously documented [1,3]. We investigated the effect of ECRT on glial cell activation within the retrosplenial cortex region because memory loss occurs following injury to this region [3,26]. Five days following 20 Gy to the hindlimb, there was a significant upregulation of the SPP1 (Table S2 and Figure 6M) protein in the retrosplenial cortex compared to that of the control mice. At 12 days following 20 Gy, there were significant upregulations of the CD11b (Table S2 and Figure 6B), CD39 (Table S2 and Figure 6D), CD9 (Table S2 and Figure 6E), GPNMB (Table S2 and Figure 6G), Ki-67 (Table S2 and Figure 6H), MAP2 (Table S2 and Figure 6I), and MHCII (Table S2 and Figure 6J) proteins compared to those of the controls. At 25 days post treatment, the retrosplenial cortex from the mice treated with 20 Gy had upregulations of the CD163 (Table S2 and Figure 6C), GPNMB (Table S2 and Figure 6G), and Ki-67 (Table S2 and Figure 6H) proteins compared to those of the control mice. 

At 6 h following 30 Gy to the hindlimb, there were significant upregulations of Aldh1l1 (Table S2 and Figure 6A) and GFAP (Table S2 and Figure 6F) proteins compared to those of the control mice. At 24 h post treatment, the Aldh1l1 (Table S2 and Figure 6A) protein remained significantly upregulated compared to that of the control mice. At 5 days post 30 Gy, there was significant upregulation of the Aldh1l1 (Table S2 and Figure 4A), GFAP (Table S2 and Figure 6F), S100B (Table S2 and Figure 6L), SPP1 (Table S2 and Figure 6M), and synaptophysin (Table S2 and Figure 6N) proteins compared to those of the control mice. At 12 days, the mice had significant upregulation of the MAP2 (Table S2 and Figure 6I) and MHCII (Table S2 and Figure 6J) and downregulation of the NeuN (Table S2 and Figure 6K) and TMEM119 (Table S2 and Figure 6O) proteins compared to those of the control mice. Finally, at 25 days following 30 Gy, the retrosplenial cortex had a significant upregulation of the Aldh1l1 (Table S2 and Figure 6A) protein compared to that of the control mice. 

### 2.5. Extracranial Causes Significant Glial Cell Activation in the Hippocampus

**Figure 7:**
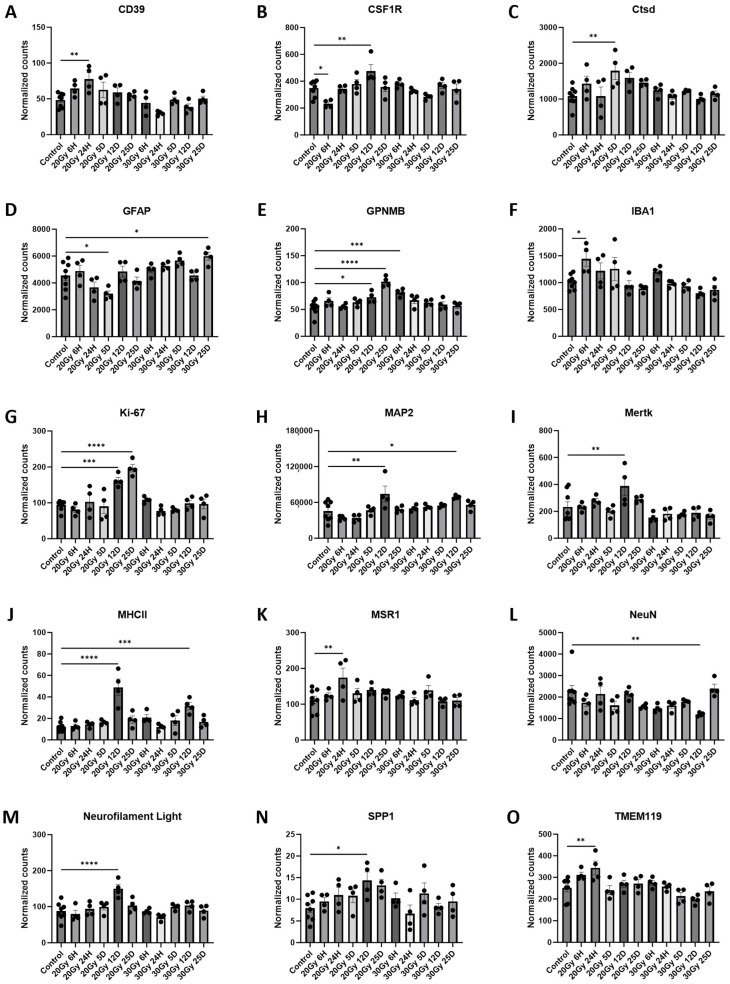

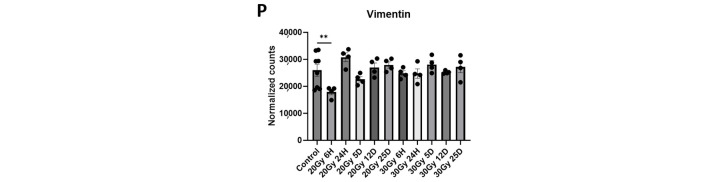
Glial cell activation in the hippocampus of ECRT-treated mice. Hippocampus from mice treated with 20 Gy demonstrated significant upregulation of CD39 (**A**), CSF1R (**B**), Ctsd (**C**), GPNMB (**E**), IBA1 (**F**), Ki-67 (**G**), MAP2 (**H**), Mertk (**I**), MHCII (**J**), MSR1 (**K**), neurofilament light chain (**M**), SPP1 (**N**), and TMEM119 (**O**). Mice treated with 20 Gy demonstrated significant downregulation of CSF1R (**B**), GFAP (**D**), and Vimentin (**P**). Hippocampus from mice treated with 30 Gy demonstrated significant upregulation of GFAP (**D**), GPNMB (**E**), MAP2 (**H**), and MHCII (**J**). Mice treated with 30 Gy demonstrated significant downregulation of NeuN (**L**). Data represent the mean ± SEM and black dots represent individual values (*n* = 4–8 mice per group). Control = control mice that were euthanized at 6 h and 25 days. 20 Gy RT = mice treated with 20 Gy radiation to hindlimb; 30 Gy RT = mice treated with 30 Gy radiation to hindlimb. * *p* < 0.05; ** *p* < 0.01; *** *p* = 0.01–0.001; **** *p* < 0.001.

The hippocampus is involved with integral aspects of memory and learning, which are critical functions that are disrupted after ECRT [3,27]. Previous studies have shown that ECRT causes upregulations of both the IBA1 and GFAP proteins within the hippocampus of mice [1,3]. Therefore, we wanted to investigate the effect of ECRT on additional hippocampal glial cell activation proteins. In the hippocampus six hours post 20 Gy, significantly increased IBA1 (Table S3 and Figure 7F) proteins and significantly decreased CSF1R (Table S3 and Figure 7B) and Vimentin (Table S3 and Figure 7P) proteins were measured compared to those of the control mice. At 24 h post 20 Gy, the mice had significant upregulations of the CD39 (Table S3 and Figure 7A), MSR1 (Table S3 and Figure 7K), and TMEM119 (Table S3 and Figure 7O) proteins compared to those of the control mice. At 5 days post 20 Gy, the mice had a significant upregulation of the Ctsd (Table S3 and Figure 7C) protein and significant downregulation of the GFAP (Table S3 and Figure 7D) protein compared to those of the control mice. Significant increases in the CSF1R (Table S3 and Figure 5B), GPNMB (Table S3 and Figure 7E), Ki-67 (Table S3 and Figure 7G), MAP2 (Table S3 and Figure 7H), Mertk (Table S3 and Figure 7I), MHCII (Table S3 and Figure 7J), neurofilament light chain (Table S3 and Figure 7M), and SPP1 (Table S3 and Figure 7N) proteins were measured 12 days post 20 Gy compared to the control. At 25 days post 20 Gy, the mice had upregulations of the GPNMB (Table S3 and Figure 7E) and Ki-67 (Table S3 and Figure 7G) proteins compared to those of the control mice. 

The hippocampus tissue from the mice treated with 30 Gy similarly had changes in multiple protein levels compared to those of the control tissue. A significant upregulation of the GPNMB (Table S3 and Figure 7E) protein was measured 6 h post 30 Gy compared to that of the control mice. At 12 days post 30 Gy, the mice had significant upregulations of the MAP2 (Table S3 and Figure 7H) and MHCII (Table S3 and Figure 7J) proteins and a significant downregulation of the NeuN (Table S3 and Figure 7L) protein compared to those of the control mice. There was a significant upregulation of the GFAP (Table S3 and Figure 7D) protein at 25 days post 30 Gy compared to that of the control mice. 

### 2.6. Extracranial Radiation Causes Molecular Changes in the Normal Brain

Neuroinflammation is associated with cognitive impairment and markers of neuron survival in cancer patients after cancer therapy [28]. We used the Nanostring nCounter^®^ (Seatle, WA, USA) glial profiling panel and neuroinflammation panel to characterize the gene expression patterns related to glial cell activation and neuroinflammation in the brains of the control mice compared to those of the mice treated with 20 Gy or 30 Gy to one hindlimb. Unsupervised hierarchical clustering of the normalized gene expression data for all the mice (Figure S1A,B) and the heat map of gene pathway cluster scores (Figure S1C,D) demonstrate distinct changes in the mice treated with either 20 Gy or 30 Gy. 

**Figure 8:**
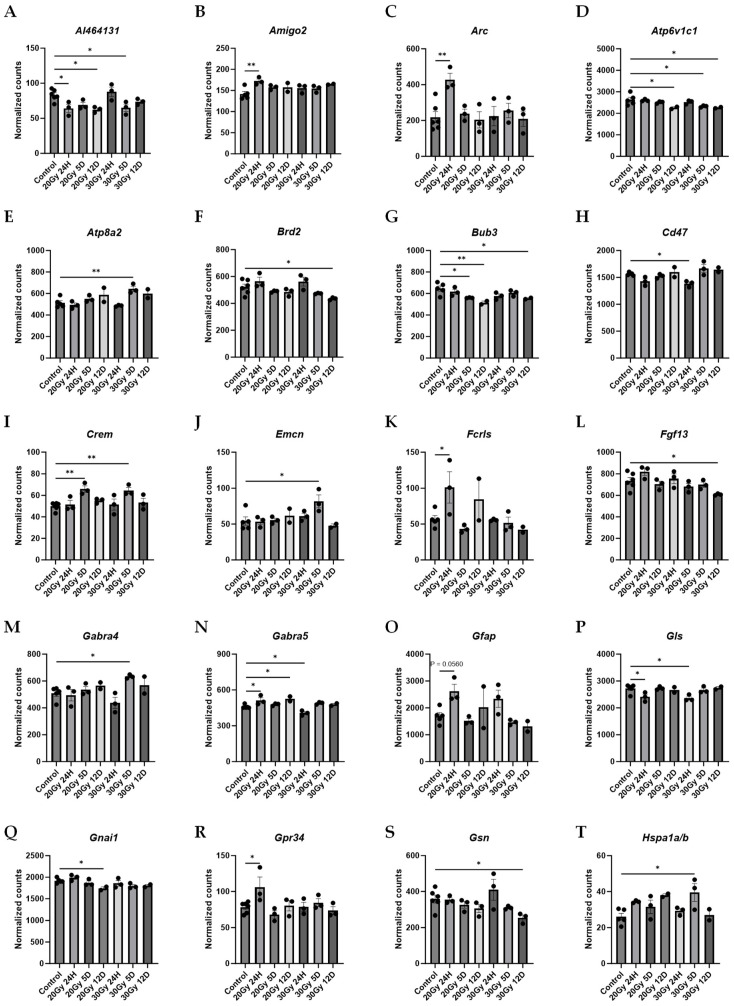

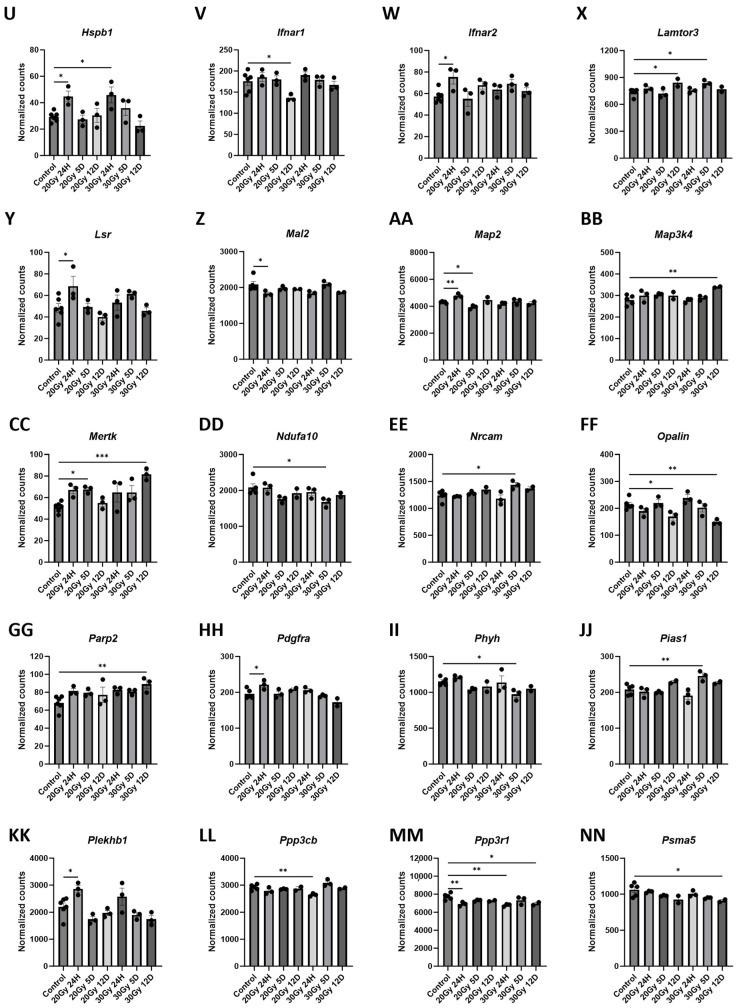

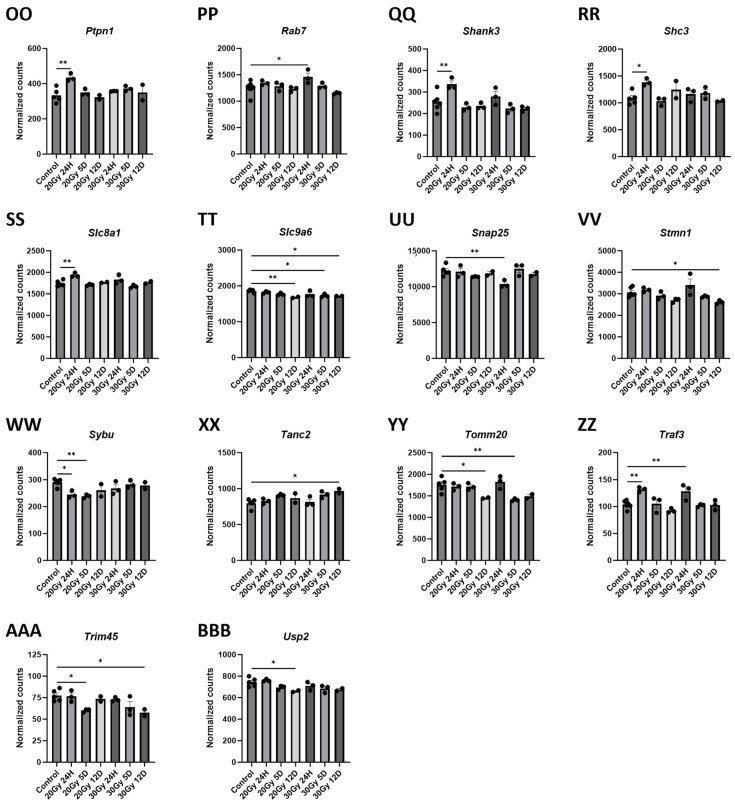
Extracranial radiation causes significant gene changes in the brain of mice. Mice treated with 20 Gy demonstrated significant upregulation of Amigo2 (**B**), Arc (**C**), Crem (**I**), Fcrls (**K**), Gabra5 (**N**), Gfap (**O**), Gpr34 (**R**), Hspb1 (**U**), Ifnar2 (**W**), Lamtor3 (**X**), Lsr (**Y**), Map2 (**AA**), Mertk (**CC**), Pdgfra (**HH**), Plekhb1 (**KK**), Ptpn1 (**OO**), Shank3 (**QQ**), Shc3 (**RR**), Slc8a1 (**SS**), and Traf3 (**ZZ**). Mice treated with 20 Gy demonstrated significant downregulation of Al464131 (**A**), Atp6v1c1 (**D**), Bub3 (**G**), Gls (**P**), Gnai1 (**Q**), Ifnar1 (**V**), Mal2 (**Z**), Map2 (**AA**), Opalin (**FF**), Ppp3r1 (**MM**), Slc9a6 (**TT**), Sybu (**WW**), Tomm20 (**YY**), Trim45 (**AAA**), and Usp2 (**BBB**). Mice treated with 30 Gy demonstrated significant upregulation of Atp8a2 (**E**), Crem (**I**), Emcn (**J**), Gabra4 (**M**), Hspa1a/b (**T**), Hspb1 (**U**), Lamtor3 (**X**), Map3k4 (**BB**), Mertk (**CC**), Nrcam (**EE**), Parp2 (**GG**), Pias1 (**JJ**), Rab7 (**PP**), Tanc2 (**XX**), and Traf3 (**ZZ**). Mice treated with 30 Gy demonstrated significant downregulation of Al464131 (**A**), Atp6v1c1 (**D**), Brd2 (**F**), Bub3 (**G**), Cd47 (**H**), Fgf13 (**L**), Gabra5 (**N**), Gls (**P**), Gsn (**S**), Ndufa10 (**DD**), Opalin (**FF**), Phyh (**II**), Ppp3cb (**LL**), Ppp3r1 (**MM**), Psma5 (**NN**), Slc9a6 (**TT**), Snap25 (**UU**), Stmn1 (**VV**), Tomm20 (**YY**), and Trim45 (**AAA**). Data represent the mean ± SEM and black dots represent individual values (*n* = 2–3 mice per group). Control = control mice that were euthanized at 24 h and 12 days. 20 Gy RT = mice treated with 20 Gy radiation to hindlimb; 30 Gy RT = mice treated with 30 Gy radiation to hindlimb. * *p* < 0.05; ** *p* < 0.01; *** *p* = 0.01–0.001. NanoString nCounter^®^ glial profiling panel and neuroinflammation panel.

The brain tissue from the mice 24 h post 20 Gy demonstrated significant upregulations in Amigo2 (Table S4 and Figure 8B), Arc (Table S4 and Figure 8C), Fcrls (Table S4 and Figure 8K), Gabra5 (Table S4 and Figure 8N), Gfap (Figure 8O), Gpr34 (Table S4 and Figure 8R), Hspb1 (Table S4 and Figure 8U), Ifnar2 (Table S4 and Figure 8W), Lsr (Table S4 and Figure 8Y), Map2 (Table S4 and Figure 8AA), Pdgfra (Table S4 and Figure 8HH), Plekhb1 (Table S4 and Figure 8KK), Ptpn1 (Table S4 and Figure 8OO), Shank3 (Table S4 and Figure 8QQ), Shc3 (Table S4 and Figure 8RR), Slc8a1 (Table S4 and Figure 8SS), and Traf3 (Table S4 and Figure 8ZZ) compared to those of the control. At 24 h post 20 Gy, the brains had significant downregulations in Al464131 (Table S4 and Figure 8A), Gls (Table S4 and Figure 8P), Mal2 (Table S4 and Figure 8Z), Ppp3r1 (Table S4 and Figure 8MM) and Sybu (Table S4 and Figure 8WW) gene expressions compared to those of the control brains. The brain tissue acquired 5 days post 20 Gy demonstrated significant upregulations in Crem (Table S4 and Figure 8I) and Mertk (Table S4 and Figure 8CC) compared to those of the control mice. At 5 days post 20 Gy, the brains had significant downregulations in Bub3 (Table S4 and Figure 8G), Map2 (Table S4 and Figure 8AA), Sybu (Table S4 and Figure 8WW), and Trim45 (Table S4 and Figure 8AAA) gene expressions compared to those of the control brains. The brain tissue from 12 days post 20 Gy demonstrated significant upregulations in Gabra5 (Table S4 and Figure 8N) and Lamtor3 (Table S4 and Figure 8X) compared to those of the control. At 12 days post 20 Gy, significant downregulations in Al464131 (Table S4 and Figure 8A), Atp6v1c1 (Table S4 and Figure 8D), Bub3 (Table S4 and Figure 8G), Gnai1 (Table S4 and Figure 8Q), Ifnar1 (Table S4 and Figure 8V), Opalin (Table S4 and Figure 8FF), Slc9a6 (Table S4 and Figure 8TT), Tomm20 (Table S4 and Figure 8YY), and Usp2 (Table S4 and Figure 8BBB) gene expressions were measured, compared to those of the control brains. 

The brain tissue from the mice 24 h post 30 Gy demonstrated significant upregulations in the Hspb1 (Table S4 and Figure 8U), Rab7 (Table S4 and Figure 8PP), and Traf3 (Table S4 and Figure 8ZZ) compared to those of the control. At 24 h post 30 Gy, the brains had significant downregulations in Cd47 (Table S4 and Figure 8H), Gabra5 (Table S4 and Figure 8N), Gls (Table S4 and Figure 8P), Ppp3cb (Table S4 and Figure 8LL), Ppp3r1 (Table S4 and Figure 8MM), and Snap25 (Table S4 and Figure 8UU) gene expressions compared to those of the control brains. The brain tissue from the mice 5 days post 30 Gy demonstrated significant upregulations in Atp8a2 (Table S4 and Figure 8E), Crem (Table S4 and Figure 8I), Emcn (Table S4 and Figure 8J), Gabra4 (Table S4 and Figure 8M), Hspa1a/b (Table S4 and Figure 8T), Lamtor3 (Table S4 and Figure 8X), Nrcam (Table S4 and Figure 8EE), and Pias1 (Table S4 and Figure 8JJ) compared to those of the control mice. At 5 days post 30 Gy, the brains had significant downregulations in Al464131 (Table S4 and Figure 8A), Atp6v1c1 (Table S4 and Figure 8D), Ndufa10 (Table S4 and Figure 8DD), Phyh (Table S4 and Figure 8II), Slc9a6 (Table S4 and Figure 8TT), and Tomm20 (Table S4 and Figure 8YY) gene expressions compared to those of the control brains. The brain tissue 12 days post 30 Gy demonstrated significant upregulations in Map3k4 (Table S4 and Figure 8BB), Mertk (Table S4 and Figure 8CC), Parp2 (Table S4 and Figure 8GG), and Tanc2 (Table S4 and Figure 8XX) compared to those of the control. At 12 days post 30 Gy, there were significant downregulations in Atp6v1c1 (Table S4 and Figure 8D), Brd2 (Table S4 and Figure 8F), Bub3 (Table S4 and Figure 8G), Fgf13 (Table S4 and Figure 8L), Gsn (Table S4 and Figure 8S), Opalin (Table S4 and Figure 8FF), Ppp3r1 (Table S4 and Figure 8MM), Psma5 (Table S4 and Figure 8NN), Slc9a6 (Table S4 and Figure 8TT), Stmn1 (Table S4 and Figure 8VV), and Trim45 (Table S4 and Figure 8AAA) gene expressions compared to those of the control brains. 

### 2.7. Gene Changes Caused by Extracranial Radiation Therapy Are Associated with Neuronal Transmission

A pathway enrichment analysis was performed on the 54 differentially expressed genes in murine brain following either 20 Gy or 30 Gy ECRT compared to brains from the control, unirradiated mice (Table 1). The 40 pathway enrichments included 13 associated with neuronal transmission, 13 associated with immune cells, 8 associated with cell signal transduction, and 1 associated with cancer. Other functions included in the enrichment analysis were insulin signaling, cytoskeleton regulation, and oxidative stress, infections, and osteoclast differentiation. 

## 3. Discussion

This study demonstrated radiation dose-dependent changes in protein and gene expressions in the brains of the mice following ECRT. Differentially expressed genes in the brains of the ECRT mice were associated with neurotransmission, glial cell activation, innate immune cell signaling, MAPK signaling, lipid metabolism, cell cytoskeleton, DNA damage, cancer, and epigenetic regulation [29,30,31,32,33]. 

Our previous study demonstrated significant increases in activated microglia and astrocytes within a variety of regions of the brain of SKH1 mice after hindlimb radiation [3]. Because it is unclear how hindlimb irradiation affects brain tissue, we evaluated the activation of microglia and astrocytes within the thoracic spinal cord to determine if a similar pathology develops or if the response was unique to unirradiated brains. Interestingly, compared to those of the control mice, the mice treated with 20 Gy had a significant upregulation of the GFAP protein within the astrocytes 5 days after treatment, but this change did not occur until 25 days after treatment in the mice treated with 30 Gy. This could have been due to inflammatory mediators localizing in the irradiated skin since substantial microscopic and macroscopic skin toxicity had begun 10 days post 30 Gy. Cytokines including Il-1, Il-6, TNF-α, and TGF-β are produced by fibroblasts, keratinocytes, endothelial cells, and immune cells at the irradiated site to recruit immune cells to this site [34]. Studies in rodent skin demonstrate a dose-dependent increase in TGF-β following irradiation that lasts days following treatment [19,35]. TGF-β can recruit inflammatory cells to the skin and is involved in the tissue repair process following radiation treatment [35]. It is possible that persistent local inflammation within the skin following the 30 Gy mice caused a delay in astrocytosis in this group, compared to the mice treated with 20 Gy. 

Following 20 Gy, increased disease-associated microglia proteins, including SPP1, GPNMB, and CD9, were increased in the striatum, retrosplenial cortex, and hippocampus, all of which contribute to memory functions [26,36,37]. Increases in these proteins are also reported in neurodegenerative diseases and intracranial cancer [38,39,40,41,42]. In contrast, only GPNMB was overexpressed in the hippocampus in murine brains following 30 Gy. The brains from this group exhibited increases in astrocyte-associated proteins, including GFAP and Aldh1l1, in the striatum, retrosplenial cortex, and hippocampus, supporting astrocytic neuroinflammation as opposed to microglial inflammation [43]. These differences highlight that radiation does not uniformly induce the same out-of-field effects, and other factors (e.g., local field-related factors) may be important contributors to neuroinflammation. Further work needs to specify and verify radiation doses in the study of neuroinflammation to avoid confounding results. 

Few proteins were upregulated in both groups similarly. MHC proteins are rare in normal brains, and the MHCII protein has been shown to be upregulated with neuroinflammation and multiple neurodegenerative diseases [44]. Indeed, murine brains had significantly upregulated MHCII in the striatum, retrosplenial cortex, and hippocampus, regardless of radiation dose. Therapies that downregulate MHCII may have a role in mitigating CRCI in cancer survivors [45,46]. Microtubule-associated protein 2 (MAP2), expressed in reactive astrocytes after brain injury potentially to stabilize the cytoskeleton [47,48,49], was upregulated 20 Gy or 30 Gy in the retrosplenial cortex and hippocampus compared to those of the controls. This may be a response or repair mechanism, and future studies may examine if changes in MAP2 are associated with the duration of cognitive dysfunction. 

Proliferation, as measured by Ki-67, was upregulated within the striatum, retrosplenial cortex, and hippocampus following 20 Gy. While there is a radiation dose-dependent effect on proliferation within irradiated tumors and irradiated brains [50,51], we are the first to show an increased proliferation in distinct structural regions of an unirradiated organ. Increased Ki-67 has also been associated with Alzheimer’s disease, Down’s syndrome, and dementia [52]. Because Ki-67 can be assessed relatively inexpensively through immunohistochemical means, it may be a reasonable, measurable target to evaluate in interventional studies seeking to minimize the neuroinflammatory effects of cancer therapy. Also following 20 Gy, the neurofilament light chain (NFL) protein, a biomarker of neurodegeneration [53], was increased in the hippocampus at the time of peak dermatitis. Importantly, serum NFL may reflect histological changes in neurodegenerative diseases, suggesting a possible serum protein to measure in patients to identify the risk or severity of CRCI during and after treatment [54].

Cellular reactions within the brain are commonly measured via evaluation of neuron-specific nuclear protein (NeuN) or synaptophysin. Interestingly, decreased NeuN and increased synaptophysin occurred only in the mice treated with 30 Gy at the timepoints assessed. NeuN is a universal marker for neurons and its expression is decreased in several pathological conditions including cerebral ischemia, hypoxia, and trauma [55]. It is possible that NeuN downregulation was simply due to a high degree of astrocytosis and microgliosis as we have previously noted in these regions after ECRT [3]. Increased synaptophysin occurs in brain injury models in rodents [56] and is a marker of neuron damage [57]; it is unclear why synaptophysin was increased early (5 days following irradiation) only in mice treated with 30 Gy.

A diverse set of gene changes occurred in mice treated with either 20 Gy or 30 Gy ECRT compared to the control mice. The genes involved with microglia function including *Cd47*, *Mertk*, *Fcrls*, *Gpr34*, *Stmn1*, *Atp6v1c1*, *Hspa1a/b*, and *Usp2* were significantly different between the irradiated and control mice. The *Cd47 Mertk*, and *Stmn1* genes have been shown to be involved in microglia activation [58,59]. The changes seen in these genes in this study supports microglia activation following ECRT. Both *Fcrls* and *Gpr34* were significantly upregulated in the brain at 24 h post 20 Gy, which is the same timepoint that multiple microglia-activated proteins were upregulated. This supports that 20 Gy ECRT induces early microgliosis [42,60,61]. It is not clear why 30 Gy does not induce this same finding, although it is possible this may occur at an altered timepoint not captured. The *Atp6v1c1* gene has been shown to be downregulated in multiple neurodegenerative diseases including Alzheimer’s, Parkinson’s, and dementia [62,63]. The *Atp6v1c1* gene was downregulated in the brain following both 20 Gy and 30 Gy. Interestingly, a reduction in the expression of the *Atp6v1c1* gene in the hippocampus was reported after whole-body irradiation in mice [64], as opposed to that after focal hindlimb irradiation. *Hspa1a/b* encodes for heat shock protein 70 (HSP70-1a and HSP70-1b) that directs proteins for lysosome degradation in the microglia, and this gene has been shown to be upregulated in neurodegenerative diseases [65,66,67]. *Hspa1a/b* was significantly upregulated in murine brains following 30 Gy, which could indicate microglia cellular stress [67]. *Usp2* downregulation is associated with stress-induced spatial memory retrieval impairment in rats [68]. There was a significant decrease in *Usp2* expression within the brain post 20 Gy, which could contribute to cognitive impairment. Notably, we previously showed in separate experiments that cognitive impairment occurred in SKH1 mice 14 days post 20 Gy to the right hindlimb [3]. 

The genes involved with astrocyte function including *Amigo2*, *Gfap*, *Al464131*, *Hspb1*, and *Nrcam* were significantly changed between the mice prescribed ECRT and the control mice. *Amigo2* is an A1-neurtoxic-associated gene in astrocytes and is involved in regulating cell survival, adhesion, neurite development, axon tract formation, and angiogenesis [69,70,71]. In a prior study, mice treated with brain irradiation had a significantly upregulated *Amigo2* expression in the hippocampus from 24 h to 1 week post treatment [69]. Our data also support that the *Amigo2* upregulation is an early change, occurring 24 h post 20 Gy. Early-onset astrocytosis was also supported by increases in the *Gfap* gene expression 24 h post 20 Gy [3]. The *Al464131* gene has been shown to be downregulated in activated astrocytes in an ischemic stroke rodent model, and in this study, it was also downregulated in the brain following 20 Gy or 30 Gy, again supporting astrocyte activation [72,73]. *Hspb1* is upregulated in response to oxidative stress, brain injury, and neurodegenerative diseases [74]. This gene was upregulated in the brain following 20 Gy or 30 Gy which could indicate oxidative stress in the brain. The *Nrcam* gene is involved with GABAergic synapse transmission and was upregulated in the brain following 30 Gy which could indicate increased inhibitory GABAergic synapse transmission [75,76]. 

Genes involved with neurotransmission and neuronal development that could negatively impact memory and cognition, including *Arc*, *Atp8a2*, *Crem*, *Shank3*, *Gabra4*, *Gabra5*, *Gls*, *Gnai1*, *Ndufa10*, *Shc3*, *Slc8a1*, *Slc9a6*, *Snap25*, *Mal2*, *Map2*, and *Fgf13*, were significantly changed in the ECRT mice compared to the control mice. The *Arc* gene plays a crucial role in the synaptic plasticity of neurons and has been shown to be increased in neuron activation associated with learning and memory [77,78]. Significant *Arc* upregulation, which is suggestive of increased neuron activation [78] occurred in the brains from the mice that received 20 Gy but not 30 Gy. The *Atp8a2* gene involved in neuron development and function has been shown when overexpressed in mice to enhance neurite outgrowth in rat hippocampal neurons [79,80]. The brain expression of *Atp8a2* increased following 30 Gy ECRT, which could be a sign of neuronal response to neuroinflammation. The *Crem* gene is involved in a variety of processes in the brain including neurogenesis, the shaping of synaptic plasticity, neurodegeneration, learning, and memory [81]. The *Crem* gene was upregulated in the brains of the irradiated mice post 20 Gy or 30 Gy, further supporting that radiation induces neuronal activation. *Shank3* regulates glutamatergic synapses and with its increased expression it causes increased functional neurotransmitter release [82]. *Shank3* gene expression was significantly increased following 20 Gy, which could suggest increased neurotransmission [82]. *Gabra4* and *Gabra5* both contribute to inhibitory synapses signaling in neurons [83,84] and affect mood and memory; both Gabra4 and Gabra5 were increased after irradiation, although the former was only increased following 30 Gy. The *Gls* gene, which encodes for the glutaminase that is the main glutamate-producer enzyme in the brain, was downregulated in the mice treated with either 20 Gy or 30 Gy, similar to other findings in neurodegenerative disease [85,86]. The *Gnai1* gene plays a role in cholinergic and GABAnergic synaptic pathways [87]. At 20 Gy ECRT, the significantly downregulated *Gnai1* gene could imply ECRT effects on cholinergic synaptic transmission. The *Ndufa10* gene downregulation in the brains of patients suffering from Alzheimer’s has been correlated with a higher degree of dementia [88]. A significant downregulation of *Ndufa10* was noted following 30 Gy, which could exert a negative effect on memory. The *Shc3* gene is involved in neurotrophic pathways controlling neuronal metabolism, learning, and memory through the regulation of NMDA receptor function in the hippocampus [89]. Early increased *Shc3* following 20 Gy could be linked to the memory deficits in these mice. The upregulation of the *Slc8a1* gene expressed in neurons and in rodent models of neurodegenerative diseases has been demonstrated to mediate microglia activation [90,91]. The early, upregulated *Slc8a1* expression following 20 Gy adds further support of early-onset microglia activation. The *Slc9a6* is found to be downregulated in both Parkinson’s and Alzheimer’s disease [92,93]. This gene was downregulated following either 20 Gy or 30 Gy ECRT. *Snap25* encodes for synaptosome-associated protein 25 (SNAP-25) which facilitates the fusion of synaptic vesicles and neurotransmitter release [94]. *Snap25* deficiency has been implicated in a variety of cognitive disorders, and it was significantly decreased early post 30 Gy [95]. *Mal2* is involved in glutamatergic neurotransmission and is downregulated in the brains of patients suffering from Alzheimer’s disease [96,97,98]. *Mal2* was significantly reduced in the brains early after 20 Gy treatment which could indicate decreased glutamatergic neurotransmission. The *Map2* gene is essential for the assembly of microtubules involved in dendritic plasticity [99,100]. *Map2* expression was significantly increased early post 20 Gy. Interestingly, MAP2 protein expression was increased 12 days post 20 Gy in multiple regions of the brain, which could be related to cognitive decline since the upregulation of Map2 expression has been shown to be correlated with cognitive decline in aging rodents [100,101]. The *Fgf13* gene promotes neuronal polarization, neurite outgrowth, and neuron migration, and the loss of FGF13 is associated with a decrease in memory and learning capacity [102,103]. *FGF13* expression was significantly decreased in the brains of the mice post 30 Gy, which could be associated with a reduced memory capacity [103]. 

Collectively, our data support neuroinflammation that occurs in mice treated with localized irradiation to a distant limb. Distinct protein and gene expression changes occur early and within 30 days of radiation; these unique patterns are attributable to different radiation doses. Many proteins and genes altered in the brains from the irradiated mice are upregulated in a variety of devastating neurodegenerative diseases that affect cognitive function. Our data support combined efforts to study these signaling pathways as they contribute to memory and cognition. Limitations in this study can be addressed in future studies. First, the mice were treated with a single radiation treatment using a high dose. This resulted in significant molecular changes in the brains of these mice. While this is applicable to human patients treated with single-fraction SRS or palliative radiation to extracranial sites, many humans are treated with lower doses of radiation for multiple treatments. In future studies, we will use multidose-radiation protocols similar to those prescribed to human cancer patients, as well as low total doses to delineate dose–response relationships in particular signaling pathways. The number of mice was small due to this being a pilot study to identify specific timepoints post treatment where there were significant molecular changes in the brain. This may have led to a type II error, particularly as we identified decreases in protein and gene expression changes that did not reach significance. Finally, while whole-brain-hemisphere gene expression changes identified key alterations following ECRT, further work is needed to identify gene changes within specific anatomic regions. Future studies will use spatial transcriptomics to evaluate gene expression in specific regions of the brain involved in memory formation in ECRT-treated mice. 

## 4. Materials and Methods

### 4.1. Experimental Animals

Nine-to-thirteen-week-old female SKH1 mice were purchased from Charles River Laboratories (Wilmington, MA, USA). Female mice were used for this study since female breast cancer survivors are disproportionately affected by ECRT-induced CRCI [7,8,10,11,16,104]. The mice were assigned groups according to body weight with 4 mice per group. The mice groups are summarized in Table 2. This study was performed with approval by and in accordance with the University of Minnesota Institutional Animal Care and Use Committee (UMN-IACUC). The mice were euthanized (E) at 6 h, 24 h, 5 days, 12 days, and 25 days post irradiation by carbon dioxide proceeded by exsanguination in accordance the UMN-IACUC Criteria for Carbon Dioxide Euthanasia Guidelines. The timepoints were selected because prior work demonstrated behavioral and histopathological changes [3]. After euthanasia, the brain, thoracic spinal cord, and skin from the right hindlimb were collected from each mouse.

### 4.2. Radiation Treatment

The mice were treated with a dose of 20 Gy or 30 Gy radiation to the skin surface of the right hindlimb using 6 MeV electrons (Varian 2100 iX; Varian Medical Systems, Inc., Palo Alto, CA, USA). The dose was selected based on our prior work which demonstrated that 30 Gy induces severe dermatitis and gliosis [19] in SKH-1 mice, whereas 20 Gy is associated with low grade (hair loss) cutaneous changes [105]. The radiation treatment protocol was identical to that previously published [3]. For immobilization, the mice were administered intraperitoneal (IP) ketamine (93–95 mg/kg) and xylazine (3–5 mg/kg). The control mice were anesthetized with the same dose of xylazine and ketamine. The mice treated with 30 Gy dose of radiation developed dermatitis on day 10. Therefore, the 30 Gy 25D mice were given carprofen subcutaneously at a dose of 1 mg/mL (~8 mg/kg) starting on day 10 once a day for 4 days to treat the dermatitis. 

### 4.3. Hematoxylin and Eosin (H&E) Stain

The irradiated skin from the right hindlimb of each mouse (*n* = 4 from each group) was fixed in 10% neutral-buffered formalin and paraffin-embedded. Five-micron sagittal tissue sections were deparaffinized in xylene and then rehydrated in graded alcohol. Slides were stained with Harris Modified Hematoxylin with acetic acid (EXPREDIA, Kalamazoo, MI, USA, Cat# 7221). The slides were dipped into acid water (0.15% HCL, Acros Organics, Fair Lawn, NJ, USA, Cat# NJAC124210010) followed by running tap water. The slides were then dipped in ammonium water (2.8% of ammonium hydroxide 28–30%, Newcomer Supply, Middleton, WI, USA, Cat# 1006A). The slides were counterstained with Eosin (Leica Biosystems, Deer Park, IL, USA, Cat# 3801600). The slides were dehydrated in graded alcohol and xylene, and then, coverslips were placed using permount mounting media (Leica Biosystems, Deer Park, IL, USA, Cat# 3801731).

### 4.4. Immunohistochemistry (IHC)

The mouse thoracic spinal cords (*n* = 4 from each group) were collected, including the spinal columns, and were fixed in 10% neutral-buffered formalin and then decalcified in 10% ethylenediaminetetraacetic acid (Fisher Scientific UK, Loughborough, UK, Cat# 118430010) in 1X PBS (MP Biomedicals, Inc NO, Solon, OH, USA, Cat# 092810305) at pH 6 for 5 days. Three-four sections of the thoracic spinal cord from each mouse were paraffin-embedded and sectioned onto slides. Five-micron tissue sections on slides were baked overnight at 60 °C. Then, the tissue sections were deparaffinized in xylene and then rehydrated in graded alcohol. Antigen retrieval took place in a Biocare Decloaking Chamber (Biocare Medical, Concord, CA, USA) at 80 °C for 1 h with sodium citrate buffer at pH 6. The tissue sections were blocked with Biocare Background Punisher (Biocare Medical, Concord, CA, USA, Cat# 50-823-79) for 30 min in a humidity chamber at room temperature. The sections were immunostained for activated microglia (rabbit polyclonal anti-Iba1; Abcam, Cambridge, MA, CAT# ab178847-100UL; 1:100 in TBS) overnight and activated astrocytes (rabbit polyclonal anti-GFAP; Abcam, Cambridge, MA, USA, Cat# ab7260-50UL; 1:2500 in PBS) for 2 h. Tissue sections were subsequently stained with a secondary antibody, goat anti-rabbit Alexa-Fluor 488 (Invitrogen, Molecular Probes, Inc., Eugene, OR, USA, Cat# A11008; 1:250 in TBS). The slides were stained, omitting the primary antibody for the negative controls. The slides were counterstained with 4′,6-Diamidino-2-Phenylindole, Dihydrochloride (DAPI, Invitrogen, Molecular Probes, Inc., Eugene, OR, USA; 1:500 in PBS) for 20 min in the dark.

### 4.5. IHC Analysis

Two thoracic spinal cords from each slide were analyzed. Using an upright microscope (Olympus BX53 microscope with Olympus DP73 camera, Olympus America Inc., Center Valley, PA, USA), 4–6 consecutive, nonoverlapping, adjacent images were acquired at 200× magnification on a DAPI filter and fluorescein isothiocyanate (FITC) filter. The corresponding DAPI and FITC images were overlaid using Photoshop 2021 version 22.5.1 (Adobe, San Jose, CA, USA). The images were analyzed using ImageJ (ImageJ 1.53e, Wayne Rashband, and contributors, National Institutes of Health, Bethesda, MD, USA, https://imagej.net/ij/ 20 August 2023). The multi-point tool was used to manually count the Dapi nuclei, Iba1+ cells, and GFAP+ cells in each consecutive image to evaluate microgliosis and astrocytosis [3]. The percentage of Iba1+ cells or percentage of GFAP+ cells was calculated by the following formula: the number of Iba1+ cells or GFAP+ cells counted/the number of Dapi nuclei counted in the same image × 100. The percentage of Iba1+ cells or percentage of GFAP+ cells from each consecutive image of the spinal cord were averaged for each mouse.

### 4.6. Tissue Preparation for Nanostring GeoMx Assay

The Nanostring GeoMx Assay was used to analyze tissue from the following treatment groups (*n* = 4 from each group) and timepoints: control 6 h, control 25 day, 20 Gy 6 h, 20 Gy 24 h, 20 Gy 5 day, 20 Gy 12 day, 20 Gy 25 day, 30 Gy 6 h, 30 Gy 24 h, 30 Gy 5 day, 30 Gy 12 day, and 30 Gy 25 day. Formalin-fixed paraffin-embedded blocks were made containing the left hemisphere of brains from 4 mice from the same group. The tissues from the FFPE blocks were sectioned at a thickness of 5 microns and mounted on Superfrost Plus Microscope Slides (Fisherbrand, Pittsburgh, PA, USA, Cat# 12-550-15). The sections were placed within the central 36.2 mm × 14.6 mm area of the slides to ensure the tissues fit within the gasketed GeoMx slide holder. The mounted tissues were dried in a hood overnight at room temperature. The slides were processed and stained using the Nanostring GeoMx Protein slide preparation protocol. DEPC-treated water (ThermoFisher, Carlsbad, CA, USA, Cat# AM9922) was used to make all solutions. The slides were baked for approximately 60 min at 60 °C. After baking, the tissue was deparaffinized and rehydrated with Citrisolv (Fisher Scientific, Cat# 04-355-121), H_2_O, and EtOH. Antigen retrieval was performed with a citrate antigen retrieval buffer (Sigma-Aldrich, St. Louis, MO, USA, Cat# C9999-100 ML, pH 6.0). The slides were placed into a TintoRetriever pressure cooker (Bio SB, Goleta, CA, USA, BSB 7008) and target retrieval was performed at a high temperature and high pressure for 15 min. After the pressure was released, the slides were cooled at room temperature for 30 min. The slides were blocked with Buffer W (Nanostring Technologies, Seattle, DC, USA, Cat# 121300313) at room temperature for 1 h.

### 4.7. Probe Incubation and Morphology Marker Staining

The prepared tissues were flooded with panels of antibody probes (Mouse neural cell profiling protein core, Mouse glial cell subtyping protein module, Nanostring Technologies, Cat# GMX-PROCO-NCT-MNCP-12 & GMX-PROMOD-NCT-MGCS-12) conjugated to oligonucleotide barcodes via photocleavable linkers. Additionally, immunofluorescence was performed to label the microglia by adding a fluorescently conjugated antibody against Iba1 (Clone E4O4W; Cell Signaling Technology, Danvers, MA, USA, Cat# 17198) to the probe mix. The slides were covered overnight at 4 °C in a humidity chamber (Simport, Quebec, Canada, Cat# M920-2). Following incubation, unbound antibodies were washed away in 3 washes of 1X TBS-T for 10 min each. Post-labeling fixation was performed with a 30 min incubation with 4% paraformaldehyde. The nuclei were stained with SYTO 13 green fluorescent nucleic acid stain (5uM, ThermoFisher, Carlsbad, CA, USA, Cat# S7575). The slides were washed in 2XSSC and loaded into the GeoMx DSP instrument slide holder.

### 4.8. GeoMx DSP AOI Selection and Barcode Collection

The GeoMx DSP instrument performs multiplexed wide-field immunofluorescence microscopy and utilizes an ultraviolet (UV) laser to release oligonucleotide barcodes from a specific area of illumination (AOI). Fluorescently labeled morphology markers enable the precise selection of regions within a tissue based on fluorescent intensity. The GeoMx instrument has a complement of four fluorescence imaging channels with the following specifications (Channel, Excitation (peak/bandwidth), Emission (peak/bandwidth)): FITC, 480/28 nm, 516/23 nm; Cy3, 538/19 nm, 564/15 nm; Texas Red, 588/19 nm, 623/30 nm; Cy5, 645/19 nm, 683/30 nm. Only the selected areas were illuminated by the UV light, thereby photocleaving the linker molecule and liberating the oligonucleotide barcodes in specific regions within the regions of interest (ROI). The thresholds can be adjusted for each segment and for each fluorescent channel and corresponding fluorescently labeled morphology marker. 

The slides were loaded and scanned in the Nanostring GeoMx DSP instrument. Exposure times for each channel were optimized for each instrument run. The exposure time was set to 75 ms for the nuclei channel (FITC) and 200 ms for the Iba1 channel (Cy5). The ROIs were drawn on the resulting images, utilizing brain architecture for guidance. Circular ROIs 500 um in diameter were placed on the striatum and retrosplenial cortex, and rectangular ROIs 500 × 675 um were placed on the hippocampus. This ROI placement strategy was replicated on each tissue section. The ROIs encompassed 500–900 cells. A total of 144 AOIs were selected. Areas of illumination (AOI) were illuminated by the UV laser in the GeoMx instrument, and the photocleaved barcodes were collected and deposited into a 96-well microplate by the instrument’s onboard microfluidics system. The collection plates were sealed to prevent contamination and stored at −80 °C prior to nCounter^®^ Readout (Nanostring Technologies). The barcodes were counted by the nCounter^®^ Analysis System (Nanostring Technologies) following Nanostring’s manufacturer protocol. The counted barcodes were imported into the GeoMx DSP platform and integrated with the ROIs selected on the slides. 

### 4.9. GeoMX DSP Analysis

The mouse neural cell profiling protein core and mouse glial cell subtyping protein module included 35 proteins with 3 housekeeping proteins and 3 negative controls (Table S5). The data were analyzed on the GeoMX DSP platform (version 2.40.421, Nanostring, GEOMX-0087) [106]. The three internal housekeeping protein probes were used to normalize the raw data following the Introduction to GeoMx Normalization: Protein manual (MK2593, Nanostring). Differential expressed proteins were determined using a linear mixed model. 

### 4.10. Nanostring Gene Expression Profiling Assay

For gene expression profiling, brain tissue from the right hemisphere was processed from three mice from the following groups: Control 24 h, 20 Gy 24 h, 30 Gy 24 h, 20 Gy 5 day, 30 Gy 5 day, Control 12 day, 20 Gy 12 day, and 30 Gy 12 day. RNA was extracted using the RNeasy Plus Universal Mini kit (Qiagen, Germantown, MD, USA, Cat# 73404) according to manufacturer’s instructions, except that instead of using gDNA eliminator solution, the wash step was split into two with RWT buffer (included in the kit), and between the wash steps, on-column DNase digestion was performed. A total of 100 ng of RNA from each sample was used to evaluate its gene expression by the nCounter^®^ glial profiling panel from Nanostring (Nanostring Technologies, Seattle, WA, USA, Cat# 115000436). Three samples were removed from the analysis from the nCounter^®^ glial profiling panel due to high binding densities (over 2.8) which was causing suppressed counts in lower-expressed genes. The manufacturer recommended using half of the amount of RNA to correct for the high binding density. Therefore, a total of 50 ng of RNA from each sample was used to evaluate gene expression by the nCounter^®^ neuroinflammation panel from Nanostring (Nanostring Technologies, Cat# 115000237). The RNA was hybridized to the codesets following the manufacturer’s protocol (nCounter^®^ XT Assay User Manual, MAN-10023-11, Nanostring Technologies, Inc., Seattle, WA, USA). The nCounter^®^ Prep station 5s was then used to prepare samples post-hybridization for data collection following manufacturer’s instructions (nCounter^®^ GEN2 Prep Station User Manual, MAN-C0020-04, Nanostring Technologies, Inc., Seattle, WA, USA). The barcodes were counted in each sample using the nCounter^®^ Analysis System following the manufacturer’s directions (nCounter^®^ Analysis System User Manual, MAN-C0035-07, Nanostring Technologies, Inc., Seattle, WA, USA). 

### 4.11. Nanostring Gene Expression Profiling Analysis

The nSolver™ Analysis Software version 4.0 (Nanostring Technologies, Seattle, WA, USA) quality control parameters were used to assess the quality of imaging, binding density, positive control linearity and positive control limit of detection. All the samples analyzed for the nCounter^®^ neuroinflammation panel passed quality control [107,108]. Eleven samples in the nCounter^®^ glial profiling panel had binding density alerts, and three of the samples were removed from the analysis due to their binding densities being above 2.8. The nSolver™ Analysis Software Advanced Analysis Module (version 2.0.134) was used to analyze the gene expression data, including gene normalization [109]. The Advanced Analysis Module was used to normalize the raw gene data for each sample to the geometric mean of the endogenous housekeeping genes using the geNorm algorithm to obtain normalized gene counts [107,108].

### 4.12. Enrichr Analysis

A pathway enrichment analysis was performed using Enrichr on the genes differentially expressed in the brains of the mice treated with ECRT compared to the control mice for both the gene panels (nCounter^®^ Glial Profiling Panel and Neuroinflammation Panel) [31,32,33]. The databases included were BioPlanet 2019, WikiPathway 2023 Human, KEGG 2021 Human, and Elsevier Pathway Collection. The top ten significant p-value enrichment results were reported for each database. 

### 4.13. Data Analysis

The data were analyzed using Prism 10.1.2 (GraphPad Software, San Diego, CA, USA). Descriptive data were used to present the data as mean ± SEM. One-way analysis of variance (ANOVA) with the Dunnett post hoc test was used to evaluate differences between the control and treatment groups for the Nanostring nCounter^®^ gene panels and GeoMx protein panels. Two-way ANOVA with the Tukey post hoc test was used to evaluate differences between the groups for the GFAP and Iba1 protein expressions in the thoracic spinal cord data. 

## 5. Conclusions

The data demonstrate there is a radiation-dose-dependent glial activation profile and differentially expressed genes in the brains of mice following ECRT to the hindlimb. Future studies that investigate regional gene expression changes in the brain after ECRT may build upon the results presented here. Collectively, our findings highlight indirect neuronal transmission and glial cell activation in the pathogenesis of ECRT-related CRCI, providing possible signaling pathways for mitigation strategies. 

## Figures and Tables

**Table 1:** Pathway enrichment for the 54 genes differentially expressed in the brains of mice after extracranial radiation therapy.

Pathway Source	Pathway Name	*p*-Value	Adjusted *p*-Value	Odds Ratio	Combined Score	Genes Included in Pathway
BioPlanet 2019	MAPK signaling pathway	1.478 × 10^−7^	0.00005026	12.88	202.56	*PDGFRA*, *PPP3CB*, *PPP3R1*, *MAP2*, *STMN1*, *HSPB1*, *FGF13*, *LAMTOR3*, *MAP3K4*
WikiPathway 2023 Human	MAPK signaling pathway WP382	0.000004232	0.0007153	12.28	151.95	*PPP3CB*, *PPP3R1*, *STMN1*, *HSPB1*, *FGF13*, *LAMTOR3*, *MAP3K4*
KEGG 2021 Human	MAPK signaling pathway	0.00001354	0.001032	10.20	114.36	*PDGFRA*, *PPP3CB*, *PPP3R1*, *STMN1*, *HSPB1*, *LAMTOR3*, *MAP3K4*
KEGG 2021 Human	Glutamatergic synapse	0.00001394	0.001032	18.57	207.63	*PPP3CB*, *PPP3R1*, *SHANK3*, *GNAI1*, *GLS*
KEGG 2021 Human	Natural killer cell mediated cytotoxicity	0.00002730	0.001347	16.05	168.68	*IFNAR2*, *PPP3CB*, *PPP3R1*, *SHC3*, *IFNAR1*
WikiPathway 2023 Human	Immune response to tuberculosis WP4197	0.00002763	0.002335	61.69	647.58	*IFNAR2*, *PIAS1*, *IFNAR1*
BioPlanet 2019	Natural killer cell-mediated cytotoxicity	0.00003385	0.003487	15.32	157.67	*IFNAR2*, *PPP3CB*, *PPP3R1*, *SHC3*, *IFNAR1*
BioPlanet 2019	CD8/T cell receptor downstream pathway	0.00003400	0.003487	24.85	255.71	*IFNAR2*, *PPP3CB*, *PPP3R1*, *IFNAR1*
BioPlanet 2019	Interferon alpha signaling regulation	0.00004103	0.003487	53.27	538.12	*IFNAR2*, *PTPN1*, *IFNAR1*
WikiPathway 2023 Human	Regulatory circuits of the STAT3 signaling pathway WP4538	0.00005838	0.002459	21.48	209.43	*IFNAR2*, *PDGFRA*, *STMN1*, *IFNAR1*
WikiPathway 2023 Human	SARS coronavirus and innate immunity WP4912	0.00007173	0.002459	43.40	414.11	*IFNAR2*, *TRAF3*, *IFNAR1*
KEGG 2021 Human	JAK-STAT signaling pathway	0.00007523	0.002784	12.86	122.12	*IFNAR2*, *PDGFRA*, *PIAS1*, *IFNAR1*, *GFAP*
WikiPathway 2023 Human	Type I interferon induction and signaling during SARS-CoV-2 infection WP4868	0.00007927	0.002459	41.84	395.12	*IFNAR2*, *TRAF3*, *IFNAR1*
WikiPathway 2023 Human	Host pathogen interaction of human coronaviruses interferon induction WP4880	0.00008730	0.002459	40.40	377.58	*IFNAR2*, *TRAF3*, *IFNAR1*
KEGG 2021 Human	GABAergic synapse	0.00009775	0.002893	18.69	172.59	*GABRA5*, *GABRA4*, *GNAI1*, *GLS*
BioPlanet 2019	EGF receptor (ErbB1) signaling pathway	0.0001145	0.007789	36.61	332.19	*PTPN1*, *GSN*, *GNAI1*
WikiPathway 2023 Human	mBDNF and proBDNF regulation of GABA neurotransmission WP4829	0.0001468	0.003544	33.46	295.37	*GABRA5*, *GABRA4*, *CREM*
BioPlanet 2019	Transmission across chemical synapses	0.0001592	0.009019	10.90	95.32	*SNAP25*, *GABRA5*, *GABRA4*, *GNAI1*, *GLS*
KEGG 2021 Human	Kaposi sarcoma-associated herpesvirus infection	0.0001712	0.004223	10.72	93.00	*IFNAR2*, *PPP3CB*, *PPP3R1*, *TRAF3*, *IFNAR1*
Elsevier Pathway Collection	Cochlear hair cell synapse proteins mutations (age-related/congenital)	0.0002545	0.01874	109.55	906.71	*SNAP25*, *GNAI1*
Elsevier Pathway Collection	HRH1/3 -> synaptic transmission	0.0002545	0.01874	109.55	906.71	*SNAP25*, *GNAI1*
Elsevier Pathway Collection	AXL receptor tyrosine kinase signaling	0.0002774	0.01874	26.61	217.92	*IFNAR2*, *HSPB1*, *IFNAR1*
BioPlanet 2019	Interferon-alpha signaling pathway	0.0003175	0.01375	95.86	772.11	*IFNAR2*, *IFNAR1*
KEGG 2021 Human	Osteoclast differentiation	0.0003836	0.008110	12.89	101.42	*IFNAR2*, *PPP3CB*, *PPP3R1*, *IFNAR1*
BioPlanet 2019	Cardiac protection against reactive oxygen species	0.0003874	0.01375	85.20	669.33	*GABRA5*, *GABRA4*
Elsevier Pathway Collection	Histamine in arousal regulation	0.0003874	0.01874	85.20	669.33	*SNAP25*, *GNAI1*
BioPlanet 2019	GABA A and B receptor activation	0.0003961	0.01375	23.41	183.36	*GABRA5*, *GABRA4*, *GNAI1*
WikiPathway 2023 Human	Interferon type I signaling pathways WP585	0.0004186	0.008844	22.95	178.49	*IFNAR2*, *PIAS1*, *IFNAR1*
Elsevier Pathway Collection	Dendritic cell activation in systemic lupus erythematosis	0.0004420	0.01874	22.50	173.83	*IFNAR2*, *TRAF3*, *IFNAR1*
BioPlanet 2019	GABA (A) receptor activation	0.0004641	0.01375	76.68	588.52	*GABRA5*, *GABRA4*
Elsevier Pathway Collection	DRD2/4 -> membrane transport	0.0004641	0.01874	76.68	588.52	*SNAP25*, *GNAI1*
Elsevier Pathway Collection	Endocannabinoids role in sleep regulation	0.0004641	0.01874	76.68	588.52	*SNAP25*, *GNAI1*
Elsevier Pathway Collection	Microtubule cytoskeleton	0.0004641	0.01874	76.68	588.52	*MAP2*, *STMN1*
KEGG 2021 Human	VEGF signaling pathway	0.0005435	0.01005	20.89	157.06	*PPP3CB*, *PPP3R1*, *HSPB1*
Elsevier Pathway Collection	Dopamine mediated glutamate release/uptake circle in neuron in migraine	0.0005476	0.01965	69.70	523.47	*SNAP25*, *GLS*
Elsevier Pathway Collection	GRM2-4/6-8 (presynaptic) -> glutamate release attenuation	0.0006377	0.02060	63.89	470.09	*SNAP25*, *GNAI1*
KEGG 2021 Human	Retrograde endocannabinoid signaling	0.0006831	0.01060	11.00	80.19	*GABRA5*, *GABRA4*, *NDUFA10*, *GNAI1*
WikiPathway 2023 Human	SARS CoV 2 innate immunity evasion and cell specific immune response WP5039	0.0007549	0.01418	18.56	133.46	*IFNAR2*, *TRAF3*, *IFNAR1*
KEGG 2021 Human	Hepatitis C	0.0008517	0.01060	10.35	73.15	*IFNAR2*, *TRAF3*, *PIAS1*, *IFNAR1*
WikiPathway 2023 Human	Insulin signaling WP481	0.0009139	0.01545	10.15	71.02	*SNAP25*, *PTPN1*, *SHC3*, *MAP3K4*

**Table 2:** Mouse group names including treatment and timepoint euthanized. RT, radiation treatment.

Group	Treatment	Timepoint
Control 6 H	None	6 h
20 Gy RT 6 H	20 Gy RT	6 h
30 Gy RT 6 H	30 Gy RT	6 h
Control 24 H	None	24 h
20 Gy RT 24 H	20 Gy RT	24 h
30 Gy RT 24 H	30 Gy RT	24 h
Control 5 D	None	5 days
20 Gy RT 5 D	20 Gy RT	5 days
30 Gy RT 5 D	30 Gy RT	5 days
Control 12 D	None	12 days
20 Gy RT 12 D	20 Gy RT	12 days
30 Gy RT 12 D	30 Gy RT	12 days
Control 25 D	None	25 days
20 Gy RT 25 D	20 Gy RT	25 days
30 Gy RT 25 D	30 Gy RT	25 days

## Data Availability

The data presented in this study are openly available in the GEO repository at https://www.ncbi.nlm.nih.gov/geo/query/acc.cgi?acc=GSE264126 (accessed on 21 May 2024), https://www.ncbi.nlm.nih.gov/geo/query/acc.cgi?acc=GSE264127 (accessed on 23 May 2023), reference numbers (GSE264126, GSE264127).

## Supplementary Materials

The following supporting information can be downloaded at: https://www.mdpi.com/article/10.3390/ijms25115731/s1.
