# Supplementary material for: Longitudinal Neuropathological Consequences of Extracranial Radiation Therapy in Mice
Source: Int J Mol Sci. 2024 May 24;25(11):5731. doi: 10.3390/ijms25115731 (PMC11171684; doi:10.3390/ijms25115731)
Supplement: Supplementary file 1 [file ijms-25-05731-s001.zip › ijms-2994393-supplementary.pdf]

Table S1: Significantly ($p<0.05$) altered protein changes in the striatum of mice treated with 20Gy or 30Gy ECRT compared to control mice (6 hour and 25 day control).

Time post ECRT	Protein	Control: Mean ± SD	20Gy: Mean ± SD	30Gy: Mean ± SD	p value
6h	Aldh1l1	724.7±166.3	736.4±166.3	1040±194.8	0.0332
	Mertk	198.8±45.63	222.0±19.65	129.5±14.43	0.0311
24h	S100b	11620±3565	6822±700.7	11472±4203	0.0221
5d	Aldh1l1	724.7±166.3	847.8±53.43	1092±86.42	0.0085
	GFAP	737.5±355.4	918.9±135.2	1649±696.8	0.0010
12d	CD11b	844.6±106.9	1047±89.33	845.1±82.80	0.0221
	GPNMB	48.60±13.09	83.94±22.56	37.71±5.554	<0.0001
	Ki-67	82.40±13.34	161.7±22.26	94.08±9.703	<0.0001
	MAP2	70533±16131	99237±10851	84376±8461	0.0040
	MHCII	13.37±4.188	64.44±29.51	36.42±8.283	<0.0001;0.0074
	NeuN	1633±428.1	1812±265.3	1009±89.46	0.0130
	TMEM119	266.2±44.14	294.0±16.42	184.9±13.91	0.0323
25d	Aldh1l1	724.7±166.3	823.8±224.3	1076±97.70	0.0132
	CD163	10.68±2.074	27.67±9.540	18.88±3.351	<0.0001
	CD40	113.6±20.96	160.5±28.08	153.7±37.44	0.0215
	GFAP	737.5±355.4	782.0±92.37	1493±234.6	0.0084
	GPNMB	48.60±13.09	131.6±3.142	64.76±7.237	<0.0001
	Ki-67	82.40±13.34	241.3±12.26	96.19±20.60	<0.0001
	Synaptophysin	418.9±88.71	469.3±49.07	663.3±154.1	0.0005

Table S2: Significantly ($p<0.05$) altered protein changes in the retrosplenial cortex of mice treated with 20Gy or 30Gy ECRT compared to control mice (6 hour and 25 day control).

Time post ECRT	Protein	Control: Mean ± SD	20Gy: Mean ± SD	30Gy: Mean ± SD	p value
6h	Aldh1l1	590.7±120.3	595.4±238.1	923.6±289.7	0.0120
	GFAP	891.2±176.2	966.6±171.8	1474±498.6	0.0040
24h	Aldh1l1	590.7±120.3	522.2±102.3	873.5±98.35	0.0463

5d	Aldh111	590.7±120.3	692.4±118.6	978.2±166.2	0.0024
	GFAP	891.2±176.2	960.9±126.2	1338±279.0	0.0444
	S100B	6553±1664	6741±2165	10351±3037	0.0051
	SPP1	6.796±2.159	15.38±3.485	15.25±9.619	0.0134, 0.0155
	Synaptophysin	379.3±48.30	406.2±33.05	552.8±177.5	0.0089
12d	CD11b	602.3±40.68	719.4±32.31	638.2±62.06	0.0321
	CD39	32.85±8.961	50.32±6.651	29.99±5.143	0.0314
	CD9	312.8±54.94	412.2±21.16	369.1±35.37	0.0226
	GPNMB	54.36±4.634	77.66±6.072	39.83±4.079	0.0006
	Ki-67	76.09±20.11	141.4±22.01	77.87±25.92	0.0001
	MAP2	58233±16705	81662±9343	87431±1473	0.0048,0.0003
	MHCII	11.59±3.834	42.01±11.16	29.58±8.250	<0.0001, <0.0001
	NeuN	2118±509.9	2198±213.3	1320±193.9	0.0097
	TMEM119	263.3±42.76	211.5±23.60	183.6±18.42	0.0187
25d	Aldh111	590.7±120.3	464.0±56.82	1034±239.4	0.0004
	CD163	8.138±4.094	20.93±3.920	11.74±3.575	<0.0001
	GPNMB	54.36±4.634	108.8±9.605	54.50±14.00	<0.0001
	Ki-67	76.09±20.11	199.6±23.03	91.15±17.61	<0.0001

Table S3: Significantly ($p<0.05$) altered protein changes in the hippocampus of mice treated with 20Gy or 30Gy ECRT compared to control mice (6 hour and 25 day control).

Time post ECRT	Protein	Control: Mean ± SD	20Gy: Mean ± SD	30Gy: Mean ± SD	p value
6h	CSF1R	349.2±59.91	232.2 ±28.68	380.8±28.88	0.0128
	GPNMB	53.65±13.16	65.77±11.81	81.21±7.156	0.0006
	IBA1	1014±133.0	1440±271.5	1195±111.3	0.0102
	Vimentin	25969±6290	17906±2054	24842±1870	0.0096
24h	CD39	48.42±9.736	77.57±17.80	29.80±3.108	0.0023
	MSR1	111.8±30.86	174.1±53.65	110.8±14.89	0.0024
	TMEM119	250.5±49.49	343.7±63.10	257.7±17.89	0.0038
5d	Ctsd	1082±272.8	1789±488.3	1223±35.49	0.0029
	GFAP	4546±1051	3187±440.8	5668±449.0	0.0292

12d	CSF1R	349.2±59.91	475.4±97.92	367.5±44.63	0.0061
	GPNMB	53.65±13.16	72.68±10.50	58.91±10.45	0.0302
	Ki-67	94.40±14.04	162.2±17.57	98.98±16.79	0.0001
	MAP2	45486±16445	74141±26641	68891±2835	0.0019, 0.0159
	Mertk	232.1±110.9	388.4±143.9	188.4±45.93	0.0082
	MHCII	12.38±4.362	48.92±15.59	31.69±6.755	<0.0001, 0.0003
	NeuN	2264±783.2	2149±266.4	1194±81.96	0.0045
	Neurofilament	87.48±23.72	149.9±23.90	103.1±13.96	<0.0001
	SPP1	7.935±2.789	14.32±4.013	8.238±1.553	0.0221
25d	GFAP	4546±1051	4140±596.6	5990±603.6	0.0176
	GPNMB	53.65±13.16	101.6±9.552	56.49±9.108	<0.0001
	Ki-67	94.40±14.04	196.9±21.23	96.91±27.57	<0.0001

A

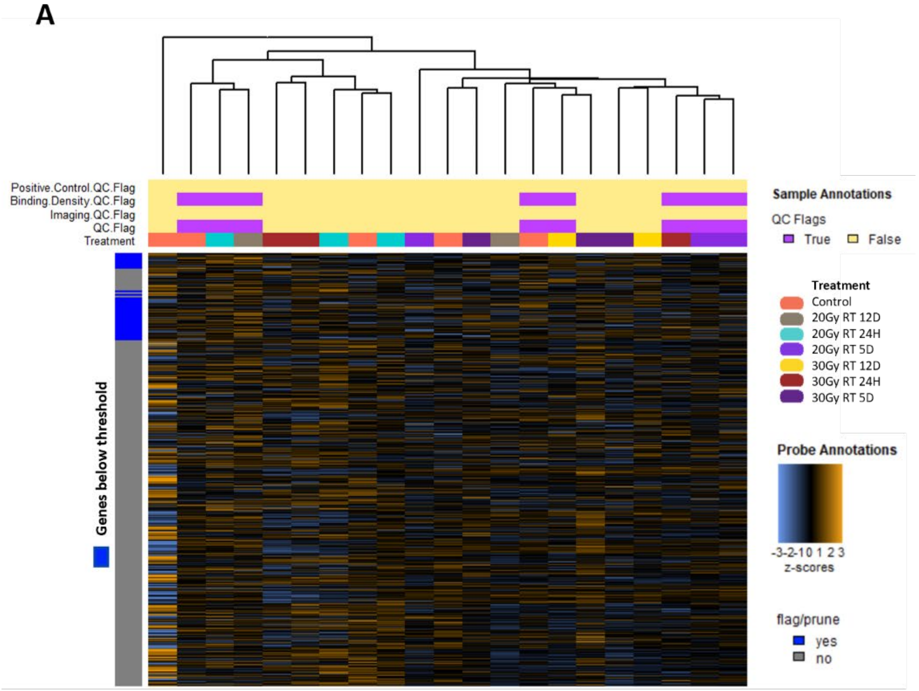
Positive.Control.QC.Flag
Binding.Density.QC.Flag
Imaging.QC.Flag
QC.Flag
Treatment
Genes below threshold
Sample Annotations
QC Flags
True
False
Treatment
Control
20Gy RT 12D
20Gy RT 24H
20Gy RT 5D
30Gy RT 12D
30Gy RT 24H
30Gy RT 5D
Probe Annotations
-3-2-10 1 2 3
z-scores
flag/prune
yes
no

B

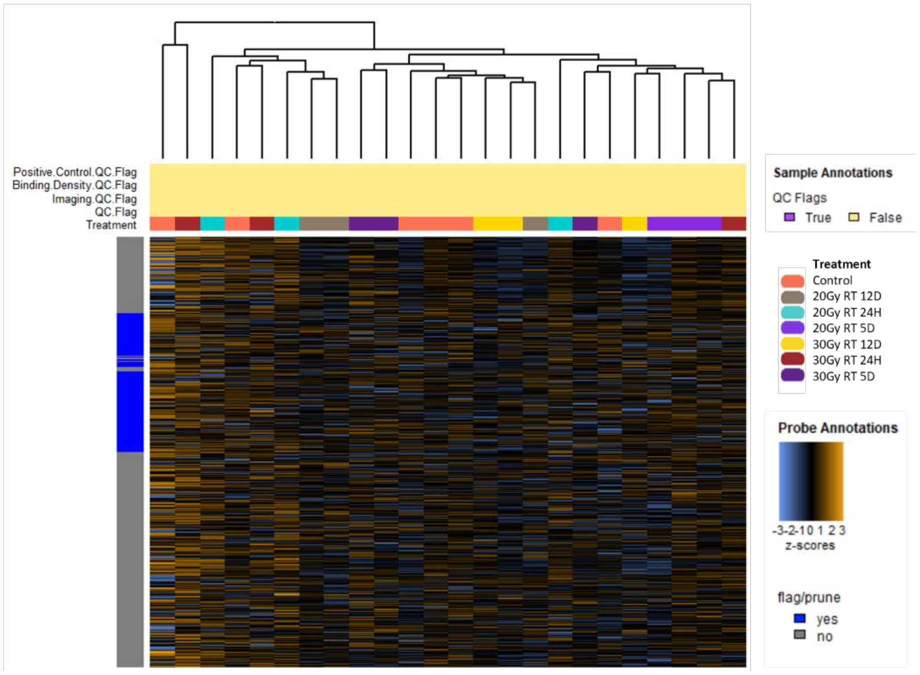
Positive.Control.QC.Flag
Binding.Density.QC.Flag
Imaging.QC.Flag
QC.Flag
Treatment
Sample Annotations
QC Flags
True
False
Treatment
Control
20Gy RT 12D
20Gy RT 24H
20Gy RT 5D
30Gy RT 12D
30Gy RT 24H
30Gy RT 5D
Probe Annotations
-3-2-10 1 2 3
z-scores
flag/prune
yes
no

C

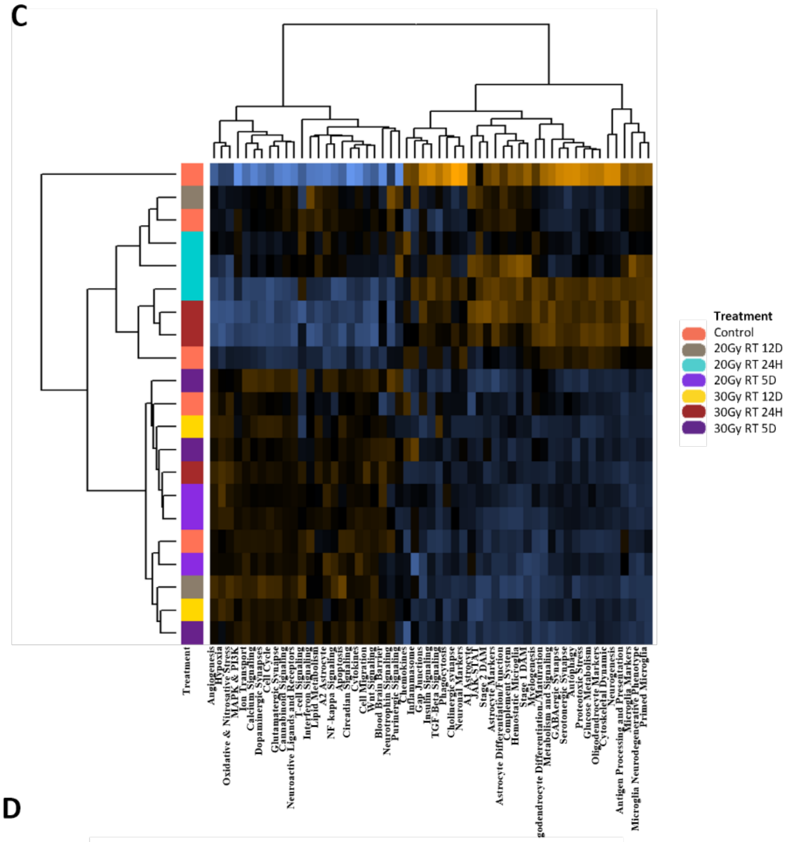
Treatment
Angiogenesis
Hypoxia
Oxidative & Nitrosative Stress
MAPK & PI3K
Ion Transport
Calcium Signaling
Dopaminergic Synapses
Cell Cycle
Glutamatergic Synapse
Cannabinoid Signaling
Neuroactive Ligands and Receptors
T-cell Signaling
Interferon Signaling
Lipid Metabolism
A2 Astrocyte
NF-kappa Signaling
Apoptosis
Circadian Signaling
Cytokines
Cell Migration
Wnt Signaling
Blood Brain Barrier
Neurotrophin Signaling
Purinergic Signaling
Chemokines
Inflammasome
Gap Junctions
Insulin Signaling
TGF-Beta Signaling
Phagocytosis
Cholinergic synapse
Neuronal Markers
A1 Astrocyte
JAK-STAT
Stage 2 DAM
Astrocyte Markers
Astrocyte Differentiation/Function
Complement System
Hemostatic Microglia
Stage 1 DAM
Myelogenesis
Metabolism and Signaling
GABAergic Synapse
Serotonergic Synapse
Autophagy
Proteotoxic Stress
Glucose Metabolism
Oligodendrocyte Markers
Cytoskeletal Dynamic
Neurogenesis
Antigen Processing and Presentation
Microglia Markers
Microglia Neurodegenerative Phenotype
Primed Microglia
Treatment
Control
20Gy RT 12D
20Gy RT 24H
20Gy RT 5D
30Gy RT 12D
30Gy RT 24H
30Gy RT 5D

D

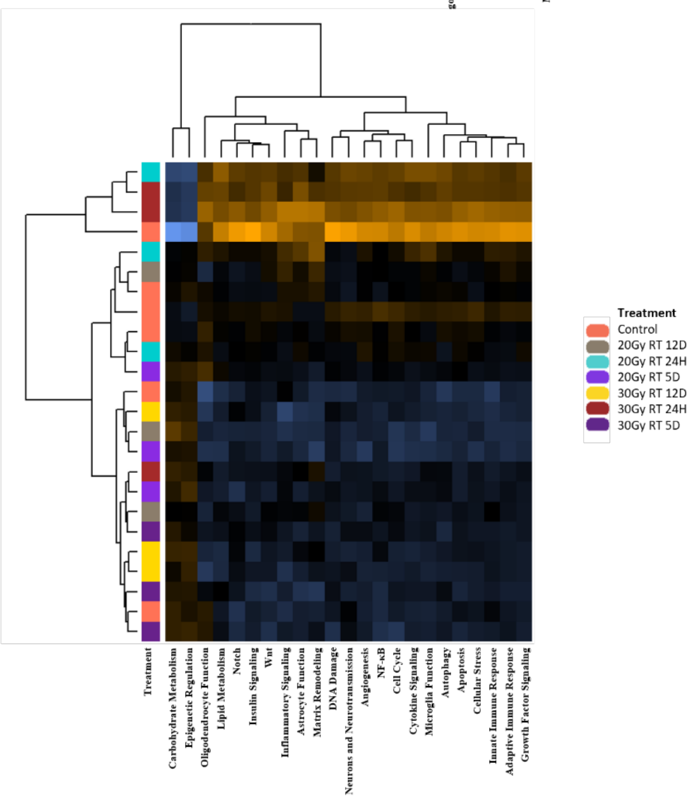
Treatment
Carbohydrate Metabolism
Epigenetic Regulation
Oligodendrocyte Function
Lipid Metabolism
Notch
Insulin Signaling
Wnt
Inflammatory Signaling
Astrocyte Function
Matrix Remodeling
DNA Damage
Neurons and Neurotransmission
Angiogenesis
NF-κB
Cell Cycle
Cytokine Signaling
Microglia Function
Autophagy
Apoptosis
Cellular Stress
Innate Immune Response
Adaptive Immune Response
Growth Factor Signaling
Treatment
Control
20Gy RT 12D
20Gy RT 24H
20Gy RT 5D
30Gy RT 12D
30Gy RT 24H
30Gy RT 5D

Figure S1: Overview of gene changes in the brain of mice after hindlimb radiation treatment. (A) Heat map of normalized data from NanoString nCounter® glial profiling panel with orange indicating high expression and blue indicating low expression. (B) Heat map of normalized data from NanoString nCounter® neuroinflammation panel with orange indicating high expression and blue indicating low expression. (C) Heatmap from NanoString nCounter® glial profiling panel showing gene pathway clustering of the groups. (D) Heatmap from NanoString nCounter® neuroinflammation panel showing gene pathway clustering of the groups. Control= control mice that were euthanized at 24 hours and 12 days. 20Gy RT= mice treated with 20Gy to hindlimb; 30Gy RT= mice treated with 30Gy to hindlimb; *n*=2-3 mice per group.

Table S4: Significantly ($p<0.05$) altered gene changes in the brain of mice treated with 20Gy or 30Gy ECRT compared to control mice (24 hour and 12 day control). G= NanoString nCounter® glial profiling panel, N= NanoString nCounter® neuroinflammation panel.

Time post ECRT	Gene	Control: Mean ± SD	20Gy: Mean ± SD	30Gy: Mean ± SD	p value
24h	Al464131(N)	83.02±7.990	64.19±9.904	87.71±11.45	0.0219
	Amigo2(G)	141.8±13.17	172.1±8.897	155.3±10.50	0.0066
	Arc(N)	218.7±75.83	427.3±62.04	224.3±93.44	0.0045
	Cd47(G)	1564±27.89	1425±81.00	1382±59.13	0.0264
	Fcrls(G)	56.85±11.02	101.2±37.86	55.72±1.495	0.0428
	Gabra5(G)	459.8±14.80	512.8±38.43	406.6±20.77	0.0192, 0.0184
	Gls(G)	2720±165.3	2407±171.6	2363±118.1	0.0354, 0.0151
	Gpr34(N)	78.05±7.555	106.2±24.13	78.58±10.70	0.0224
	Hspb1(N)	29.34±3.837	44.77±6.996	45.88±10.37	0.0382, 0.0244
	Ifnar2(N)	57.41±6.033	75.40±11.34	63.76±6.397	0.0243
	Lsr(N)	48.47±10.00	68.65±15.91	53.32±12.21	0.0381
	Mal2(G)	2082±190.2	1831±70.91	1836±70.86	0.0477
	Map2(G)	4306±65.43	4770±157.3	4150±176.5	0.0094
	Pdgfra(G)	195.5±13.19	221.2±13.08	206.2±7.310	0.0350
	Plekhb1(N)	2195±348.3	2859±226.9	2571±549.0	0.0360
	Ppp3cb(G)	2923±102.9	2787±124.0	2641±55.93	0.0054
	Ppp3r1(G)	7732±354.8	6892±236.1	6778±141.3	0.0064, 0.0023
	Ptpn1(G)	333.3±39.03	434.8±22.22	357.5±1.593	0.0021
	Rab7(N)	1252±132.0	1345±41.62	1458±132.0	0.0328
	Shank3(N)	253.5±41.88	336.1±27.53	279.4±40.52	0.0072

	Shc3(G)	1083±115.4	1383±65.85	1162±129.7	0.0150
	Slc8a1(G)	1743±67.53	1936±51.90	1833±105.0	0.0035
	Snap25(G)	12221±708.6	12082±810.5	10367±505.5	0.0066
	Sybu(G)	290.2±14.41	244.8±13.75	267.7±23.96	0.0160
	Traf3(N)	103.7±7.430	131.8±4.496	128.2±14.83	0.0023, 0.0076
5d	Al464131(N)	83.02±7.990	69.01±6.695	64.91±9.090	0.0285
	Atp6v1c1(G)	2635±250.2	2510±51.77	2323±42.70	0.0497
	Atp8a2(G)	513.4±44.23	550.3±31.42	642.9±43.38	0.0075
	Bub3(G)	646.3±53.88	559.9±3.280	604.7±28.66	0.0325
	Crem (N)	49.93±3.560	65.89±5.198	64.50±4.817	0.0040, 0.0086
	Emcn(G)	54.26±13.06	55.41±4.800	81.68±15.59	0.0185
	Gabra4(G)	507.9±49.20	534.4±41.86	632.9±14.62	0.0433
	Hspa1a/b(G)	26.01±4.152	31.48±6.447	39.49±8.643	0.0104
	Lamtor3(G)	734.3±43.09	720.9±52.53	835.1±35.14	0.0290
	Map2(G)	4306±65.43	3933±138.9	4366±221.0	0.0404
	Mertk(N)	51.16±4.664	67.55±3.311	64.73±11.04	0.0449
	Ndufa10(G)	2086±219.8	1755±97.34	1677±134.8	0.0220
	Nrcam(G)	1240±97.24	1284±34.20	1436±78.69	0.0238
	Phyh(G)	1151±54.40	1038±26.98	971.8±76.06	0.0438
	Pias1(G)	207.6±14.78	199.8±3.434	245.7±14.03	0.0078
	Slc9a6(G)	1861±40.00	1766±37.34	1728±47.00	0.0106
	Sybu(G)	290.2±14.41	238.4±6.833	282.5±14.67	0.0061
	Tomm20(G)	1756±158.6	1712±79.14	1405±30.93	0.0043
	Trim45(G)	77.54±6.609	60.06±1.965	64.13±11.19	0.0122
12d	Al464131(N)	83.02±7.990	62.47±4.116	73.67±4.319	0.0116
	Atp6v1c1(G)	2635±250.2	2251±70.97	2261±38.41	0.0327, 0.0384
	Brd2(N)	521.8±52.76	484.4±28.74	435.0±9.471	0.0386
	Bub3(G)	646.3±53.88	515.0±21.93	554.1±8.403	0.0044, 0.0494
	Fgf13(N)	735.4±74.80	703.4±49.40	608.2±8.613	0.0411
	Gabra5(G)	459.8±14.80	525.4±28.06	477.7±14.13	0.0111
	Gnai1(G)	1920±55.67	1740±53.78	1802±43.27	0.0424
	Gsn(N)	358.4±53.85	305.8±39.32	253.7±28.31	0.0463

	Ifnar1(N)	175.8±23.73	136.5±7.966	167.0±15.31	0.0304
	Lamtor3(G)	734.3±43.09	842.8±60.82	768.5±41.17	0.0423
	Map3k4(G)	279.5±22.70	299.6±24.13	339.4±3.505	0.0091
	Mertk(N)	51.16±4.664	55.00±5.379	81.59±5.385	0.0002
	Opalin(N)	214.4±18.89	169.1±22.23	148.9±9.339	0.0296, 0.0015
	Parp2(N)	68.20±8.004	77.21±14.85	89.03±8.314	0.0083
	Ppp3r1(G)	7732±354.8	7262±95.79	6933±228.3	0.0239
	Psma5(G)	1062±91.66	926.2±71.93	908.1±23.96	0.0278
	Slc9a6(G)	1861±40.00	1680±23.65	1717±0.6986	0.0027, 0.0161
	Stmn1(N)	3072±208.9	2701±99.39	2613±82.63	0.0481
	Tanc2(G)	797.8±62.93	869.5±92.09	966.4±43.83	0.0146
	Tomm20(G)	1756±158.6	1453±16.38	1491±67.72	0.0319
	Trim45(G)	77.54±6.609	73.48±4.250	57.42±5.563	0.0118
	Usp2(G)	743.9±41.44	660.3±10.88	674.9±17.06	0.0333

Table S5: Protein panel used for mouse brain protein spatialomics.

**Protein**	**Panel**	**Cell Type**
Aldh1l1	Glial Cell Subtyping	Astrocyte
CD9	Glial Cell Subtyping	Disease-Associated Microglia
CSF1R	Glial Cell Subtyping	Microglia
Ctsd	Glial Cell Subtyping	Microglia
GPNMB	Glial Cell Subtyping	Disease-Associated Microglia
ITGAX	Glial Cell Subtyping	Microglia
MSR1	Glial Cell Subtyping	Microglia
Mertk	Glial Cell Subtyping	Disease-Associated Microglia
SPP1	Glial Cell Subtyping	Disease-Associated Microglia
Vimentin	Glial Cell Subtyping	Astrocyte
CD11b	Neural Cell Profiling	DC, Myeloid, Microglia
CD163	Neural Cell Profiling	M2 Macrophage, Macrophage, Myeloid, Myeloid Suppression
CD31	Neural Cell Profiling	Endothelial
CD39	Neural Cell Profiling	Myeloid Suppression, Inflammation, Microglia
CD40	Neural Cell Profiling	Myeloid, Myeloid Activation, Microglia

CD45	Neural Cell Profiling	Leukocytes
CD68	Neural Cell Profiling	M2 Macrophage, Macrophage, Myeloid
GAPDH	Neural Cell Profiling	Housekeepers
GFAP	Neural Cell Profiling	Astrocyte, Inflammation
Histone H3	Neural Cell Profiling	Housekeepers
IBA1	Neural Cell Profiling	Microglia
Ki-67	Neural Cell Profiling	Proliferation
MAP2	Neural Cell Profiling	Cytoskeleton, Neuron
MHC II	Neural Cell Profiling	Antigen Presentation, MHC2, Microglia
Myelin basic protein	Neural Cell Profiling	Oligodendrocytes
NeuN	Neural Cell Profiling	Neuron
Neurofilament light	Neural Cell Profiling	Cytoskeleton, Neuron
Olig2	Neural Cell Profiling	Oligodendrocytes
Rb IgG	Neural Cell Profiling	Background
Rt IgG2a	Neural Cell Profiling	Background
Rt IgG2b	Neural Cell Profiling	Background
S100B	Neural Cell Profiling	Antigen, Astrocyte, Inflammation, Melanoma, Tumor
S6	Neural Cell Profiling	Housekeepers
Synaptophysin	Neural Cell Profiling	Synaptic Vesicle
TMEM119	Neural Cell Profiling	Microglia
